# Proteomic Dissection of the Cellulolytic Machineries Used by Soil-Dwelling *Bacteroidetes*

**DOI:** 10.1128/mSystems.00240-18

**Published:** 2018-11-20

**Authors:** Marcel Taillefer, Magnus Ø. Arntzen, Bernard Henrissat, Phillip B. Pope, Johan Larsbrink

**Affiliations:** a Wallenberg Wood Science Center, Department of Biology and Biotechnology, Chalmers University of Technology, Gothenburg, Sweden; b Faculty of Chemistry, Biotechnology and Food Science, Norwegian University of Life Sciences, Ås, Norway; c Architecture et Function des Macromolécules Biologiques, CNRS, Aix-Marseille University, Marseille, France; Woods Hole Oceanographic Institution

**Keywords:** carbohydrate-active enzymes, cellulase, cellulose, proteomics, soil microbiology

## Abstract

Bacteria of the phylum *Bacteroidetes* are regarded as highly efficient carbohydrate metabolizers, but most species are limited to (semi)soluble glycans. The soil *Bacteroidetes* species Cytophaga hutchinsonii and Sporocytophaga myxococcoides have long been known as efficient cellulose metabolizers, but neither species conforms to known cellulolytic mechanisms. Both species require contact with their substrate but do not encode cellulosomal systems of cell surface-attached enzyme complexes or the polysaccharide utilization loci found in many other *Bacteroidetes* species. Here, we have fractionated the cellular compartments of each species from cultures growing on crystalline cellulose and pectin, respectively, and analyzed them using label-free quantitative proteomics as well as enzymatic activity assays. The combined results enabled us to highlight enzymes likely to be important for cellulose conversion and to infer their cellular localization. The combined proteomes represent a wide array of putative cellulolytic enzymes and indicate specific and yet highly redundant mechanisms for cellulose degradation. Of the putative endoglucanases, especially enzymes of hitherto-unstudied glycoside hydrolase family, 8 were abundant, indicating an overlooked important role during cellulose metabolism. Furthermore, both species generated a large number of abundant hypothetical proteins during cellulose conversion, providing a treasure trove of targets for future enzymology studies.

**IMPORTANCE** Cellulose is the most abundant renewable polymer on earth, but its recalcitrance limits highly efficient conversion methods for energy-related and material applications. Though microbial cellulose conversion has been studied for decades, recent advances showcased that large knowledge gaps still exist. Bacteria of the phylum *Bacteroidetes* are regarded as highly efficient carbohydrate metabolizers, but most species are limited to (semi)soluble glycans. A few species, including the soil bacteria C. hutchinsonii and S. myxococcoides, are regarded as cellulose specialists, but their cellulolytic mechanisms are not understood, as they do not conform to the current models for enzymatic cellulose turnover. By unraveling the proteome setups of these two bacteria during growth on both crystalline cellulose and pectin, we have taken a significant step forward in understanding their idiosyncratic mode of cellulose conversion. This report provides a plethora of new enzyme targets for improved biomass conversion.

## Abstract (precis)

Cellulose is the most abundant renewable polymer on earth, but its recalcitrance limits highly efficient conversion methods for energy-related and material applications. Though microbial cellulose conversion has been studied for decades, recent advances showcased that large knowledge gaps still exist. Bacteria of the phylum *Bacteroidetes* are regarded as highly efficient carbohydrate metabolizers, but most species are limited to (semi)soluble glycans. A few species, including the soil bacteria C. hutchinsonii and S. myxococcoides, are regarded as cellulose specialists, but their cellulolytic mechanisms are not understood, as they do not conform to the current models for enzymatic cellulose turnover. By unraveling the proteome setups of these two bacteria during growth on both crystalline cellulose and pectin, we have taken a significant step forward in understanding their idiosyncratic mode of cellulose conversion. This report provides a plethora of new enzyme targets for improved biomass conversion.

## INTRODUCTION

Production of biofuels and other commodities from renewable sources has steadily increased in importance as environmentally favorable alternatives to fossil-derived products. First-generation biofuel production, i.e., biofuel derived from starch and sucrose, has been criticized with respect to potential socioeconomic and environmental issues due to direct competition with land usage for crop growth (1), and further development of second-generation biofuels from lignocellulosic biomass is desirable (2). Lignocellulose primarily consists of cellulose, hemicelluloses, and lignin, where cellulose represents the most abundant polymer, though its crystalline nature makes it highly recalcitrant to enzymatic degradation. Microorganisms have evolved complex systems to degrade cellulose: noncomplexed enzyme systems involving the secretion of cellulases into the environment as well as complexed systems involving (cell surface-attached) multienzyme complexes (cellulosomes) (3, 4). Both of these strategies are assumed to require both β-1,4-endoglucanases (EGs) (to cleave amorphous cellulose chains) and *exo*-acting cellobiohydrolases (CBHs), which attack the cellulose chains from either the reducing end or the nonreducing end (5–7). Product inhibition of EGs and CBHs by the main product, cellobiose, is alleviated by β-glucosidases (BGs), which cleave di- and oligosaccharides into glucose. In the Carbohydrate-active enzymes database CAZy (www.cazy.org [8]), EGs are typically found in glycoside hydrolase (GH) families GH5 and GH9; CBHs in GH6, GH7, and GH48; and BGs in GH1 and GH3. The discovery of lytic polysaccharide monooxygenases (LPMOs) that cleave glycosidic bonds via oxidation and substantially boost the action of the aforementioned hydrolytic enzymes has greatly improved our understanding of microbial turnover of lignocellulose (9–13).

Cytophaga hutchinsonii and Sporocytophaga myxococcoides are two Gram-negative, aerobic, mesophilic, cellulose-degrading bacterial species belonging to the *Bacteroidetes* phylum (14–17). Both species were isolated almost a century ago but remain poorly studied, and their mechanisms of carbon source utilization are unclear. A striking feature of C. hutchinsonii and S. myxococcoides is their ability to efficiently grow on crystalline cellulose despite an apparent lack of both CBHs and LPMOs in their respective genomes (14–16, 18–20). While both species encode several EGs, these apparently do not account for their efficient cellulose utilization (19–22). Further, their cellulolytic machineries are known to be cell associated but do not contain any cellulosome-associated dockerin or scaffoldin proteins (14, 18, 20, 23–25). In several *Bacteroidetes* species, so-called polysaccharide utilization loci (PULs) encode the required proteins and enzymes for degradation, capture, and import of targeted carbohydrates, both soluble and crystalline (26–28). In C. hutchinsonii, a protein pair homologous to the canonical Bacteroides thetaiotaomicron SusC/D proteins (sugar transport and capture; PUL identifiers), is found in the genome, though disruption of the encoding genes did not result in an observable phenotype regarding growth on cellulose (29), indicating that PULs are not used for this process.

Both C. hutchinsonii and S. myxococcoides are viewed as cellulose specialists, as they are extremely limited in their carbon source utilization profiles. Apart from cellulose, both species can utilize cellobiose and glucose, but this requires extensive adaption periods and, likely, mutational events (30, 31). S. myxococcoides has been shown to disrupt the hemicellulose xylan, though it is incapable of utilizing either the polysaccharide or its main constituent xylose as the sole carbon source (30, 32). Both species are immobile in liquid cultures but exhibit rapid gliding motility on solid surfaces similar to that seen with the more extensively studied *Bacteroidetes* species Flavobacterium johnsoniae (14, 15, 17, 20, 33). Many genes associated with motility and polysaccharide degradation in F. johnsoniae are linked to its type 9 secretion system (T9SS). The T9SS in C. hutchinsonii is also essential in polysaccharide utilization, as mutating the T9SS *sprP* component abolishes the ability to utilize crystalline cellulose (34). Further, many genes putatively related to cellulose utilization in C. hutchinsonii contain carboxy-terminal domains (CTDs; TIGRFAMs TIGR04183 and TIGR04131), which direct the proteins for transport through the outer membrane by the T9SS (35, 36), and a strong link can be postulated between the T9SS and motility and cellulose utilization in both C. hutchinsonii and S. myxococcoides.

In previous investigations of C. hutchinsonii, individual cellulases were identified and studied through mutagenesis and biochemical characterization (22, 37–42). The mode of cellulose turnover by this bacterium remains enigmatic, however, and similar studies of S. myxococcoides are nonexistent. As the cellulolytic systems of both species may encompass industrially relevant enzymes and/or present a new paradigm in microbial cellulose turnover, we chose to investigate the entire proteomes of both species during growth. Proteomic analyses were performed on different cellular compartments during both early and late growth on cellulose as well as pectin as a non-cellulosic/glucose-based control, and the analyses were further coupled to enzyme activity analyses.

## RESULTS

### Carbohydrate utilization and carbohydrate-active enzyme repertoires.

The carbohydrate-active enzyme (CAZyme) repertoire of C. hutchinsonii is available in the CAZy database (see Table S1 in the supplemental material) and consists of 189 unique proteins. The genome of S. myxococcoides was analyzed using the CAZy pipeline (8), and the presence of 289 putative CAZymes, including putative cellulases (GH5, GH8, and GH9), was revealed (Table S2). As expected, no putative CBHs or LPMOs were identified. S. myxococcoides encodes eight putative BGs (GH1 and GH3), which were predicted to be intracellular. The bacterium encodes nine putative GH5 enzymes (C. hutchinsonii encodes five); all but one of these were predicted to be extracellular enzymes. Also, genes encoding putative GH8 and GH9 enzymes were more numerous in the S. myxococcoides genome (11 GH8-encoding genes and 8 GH9-encoding genes) than in the C. hutchinsonii genome (6 GH8-encoding genes and 7 GH9-encoding genes). Despite the presence of multiple GH8 members, the possible involvement of GH8 enzymes in cellulose conversion had not been reported in previous studies of C. hutchinsonii (20, 43). The GH8 enzymes of both species were predicted to be either extracellular and soluble or localized in the outer membrane, whereas the GH9 enzymes were predicted to be either extracellular or periplasmic. S. myxococcoides also encodes multiple putative hemicellulose- and pectin-modifying enzymes (Table S2).

10.1128/mSystems.00240-18.4 TABLE S1 Abundance and localization of putative CAZYmes in C. hutchinsonii. The predicted localization is based on prediction using pSORTb 3.0 (65). The proteome localizations were based on overrepresentation in a fraction and are abbreviated as follows: C, cytoplasmic; P, periplasmic; S, secreted extracellular soluble; IM, inner membrane; OM, outer membrane; L, lipoprotein. The presence of the T9SS was based on the presence of the TIGR04183 or TIGR04131 carboxy-terminal domain. Red shading represents log_10_ LFQ intensity levels over 6, purple levels between 5.5 and 5.99, blue levels between 5 and 5.49, and turquoise levels between 4.5 and 4.99. A lack of shading indicates that the levels of the proteins in question were below detectable limits. Download Table S1, DOCX file, 0.04 MB. Copyright © 2018 Taillefer et al. 2018 Taillefer et al. This content is distributed under the terms of the Creative Commons Attribution 4.0 International license.

10.1128/mSystems.00240-18.5 TABLE S2 Abundance and localization of putative CAZYmes in S. myxococcoides. Predicted functions of the enzyme families or binding targets for CBMs are indicated. The predicted localization is based on prediction using pSORTb 3.0 (65). The proteome localizations were based on overrepresentation in a fraction and are abbreviated as follows: C, cytoplasmic; P, periplasmic; S, secreted extracellular soluble; IM, inner membrane; OM, outer membrane; L, lipoprotein. The presence of the T9SS was based on the presence of the TIGR04183 or TIGR04131 carboxy-terminal domain. Red shading represents log_10_ LFQ intensity levels over 8.5, purple levels between 7.75 and 8.49, blue levels between 7 and 7.74, turquoise levels between 6.5 and 6.99, and green levels between 5.5 and 6.49. A lack of shading indicates that the levels of the proteins in question were below detectable limits. Download Table S2, DOCX file, 0.04 MB. Copyright © 2018 Taillefer et al. 2018 Taillefer et al. This content is distributed under the terms of the Creative Commons Attribution 4.0 International license.

**FIG 1:**
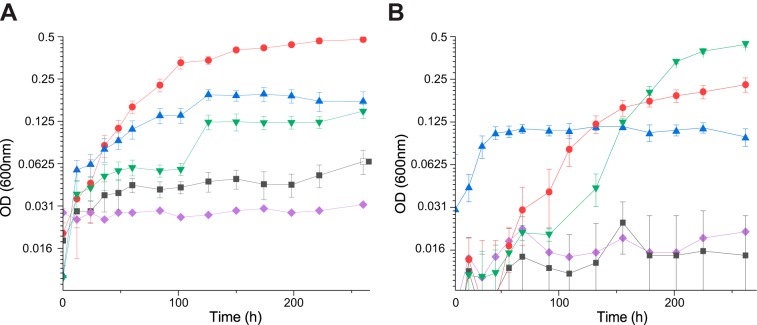
Semi-log growth curves of C. hutchinsonii (A) and S. myxococcoides (B) in MMM with cellobiose (■), glucose (●), pectin (▲), mannose (▼), or no carbon source (♦). Error bars represent standard deviations of results from triplicate experiments. OD, optical density.

While both C. hutchinsonii and S. myxococcoides are regarded as cellulose specialists, their respective encoded abundances of CAZymes indicate that they possess the ability to deconstruct various polysaccharides. No comprehensive growth data on polysaccharides other than cellulose are available in the literature; thus, both species were assayed for growth on multiple mono- and polysaccharides. Both C. hutchinsonii and S. myxococcoides were able to grow on filter paper (20, 37, 43), pectin, and mannose. To our knowledge, this represents the first reported study of C. hutchinsonii or S. myxococcoides growing on any polysaccharide other than cellulose. The C. hutchinsonii strain used (G2, glucose adapted) was, as expected, able to grow on glucose, but S. myxococcoides exhibited a lag phase of several days before growing on this monosaccharide (Fig. 1) (30, 31). Interestingly, despite the ability to metabolize mannose, neither organism was able to utilize mannan polysaccharides (galactomannan and glucomannan), and both were further unable to utilize xylose, xylan, or xyloglucan as carbon sources, despite the β-1,4-glucan backbone of the latter. During growth on mannose, curiously, S. myxococcoides formed yellow spheres, which later disintegrated, leading to a sudden increase in turbidity (Fig. 1). The growth of both species on filter paper (crystalline cellulose) led to initial colonization of the substrate without an apparent increase in the turbidity of the liquid media due to the strong cell attachment to the solid cellulose filaments. Growth on the solid substrate continued until the paper strips disintegrated, causing the cells to be released into the medium in a manner analogous to previously reported data on filter paper utilization (37, 43). Regardless of the carbon source, both C. hutchinsonii and S. myxococcoides grew relatively slowly in liquid media but exhibited rapid growth on agar plates overlaid with filter paper strips.

### Analysis of the C. hutchinsonii proteome.

The proteome of C. hutchinsonii was obtained from cells grown with either filter paper (cellulose) or pectin (non-glucose-based control) as the sole carbon source, from both early and late growth. During the time period of late growth on filter paper, the cellulose had visibly disintegrated, with the bacteria being in solution rather than surface attached. In addition to the total proteome, proteins from the late growth stages were fractionated to investigate protein abundances in different cellular compartments, including the abundances of extracellular soluble, periplasmic, inner membrane, and outer membrane proteins. The fractionation procedure and protein cellular localizations were verified by enrichment of homologues of the marker proteins ompA (outer membrane protein A), outer membrane (44); ptsS (phosphate ABC transporter phosphate-binding protein), periplasm (45); and nuoL (NADH-quinone oxidoreductase subunit L), inner membrane (46) (Table S3). The abundances of individual proteins were determined using label-free quantitative (LFQ) proteomic analyses (47). The total number of detected proteins from cells grown on filter paper (1,301 proteins) was much greater than the number from pectin-grown cells (337 proteins); similarly, the total number of CAZymes detected was much larger in filter paper-grown cells (50 proteins) than in pectin-grown cells (11 proteins) (Table S1; see also Fig. S1 in the supplemental material).

10.1128/mSystems.00240-18.1 FIG S1 Heatmap of the predicted carbohydrate active enzymes from C. hutchinsonii detectable during growth on filter paper and/or pectin. Download FIG S1, EPS file, 1.3 MB. Copyright © 2018 Taillefer et al. 2018 Taillefer et al. This content is distributed under the terms of the Creative Commons Attribution 4.0 International license.

10.1128/mSystems.00240-18.6 TABLE S3 Percent total log_10_ LFQ intensities of the cell localization markers in both C. hutchinsonii and S. myxococcoides. ompA (outer membrane protein A), outer membrane; ptsS (phosphate ABC transporter phosphate-binding protein), periplasm; nuoL (NADH-quinone oxidoreductase subunit L), inner membrane. Download Table S3, DOCX file, 0.01 MB. Copyright © 2018 Taillefer et al. 2018 Taillefer et al. This content is distributed under the terms of the Creative Commons Attribution 4.0 International license.

### Endoglucanases.

The five putative GH5 enzymes encoded by C. hutchinsonii were all identified in the proteome in the early stage of growth on filter paper; three of the five (locus tag identifiers C. hutchinsonii 1107 [CHU_1107], CHU_1842, and CHU_2149) remained in the late stage of growth (Table 1; see also Fig. S1). Two enzymes, CHU_1107 and CHU_1727, were also present in both the early and late growth stages on pectin. Based on abundances in the cellular fractions, both CHU_1107 and CHU_1842 were predicted to be inner membrane-bound proteins despite CHU_1107 containing a CTD. Further, CHU_2103 and CHU_2149 were associated with the outer membrane whereas CHU_1727 was abundant in the extracellular soluble fraction. CHU_2103 has been shown to be abundant in membrane enrichments and soluble when cell lysates were separated (37), which, together with the proteomic data, suggests that CHU_2103 is an outer membrane protein facing the periplasm. CHU_2103 mutants have shown moderate reductions in the ability to digest crystalline cellulose (22, 37, 48). Our observation that CHU_2103 was present only when cells were attached to solid filter paper indicates a role in crystalline cellulose conversion (Table 1; see also Fig. S1).

**TABLE 1:**
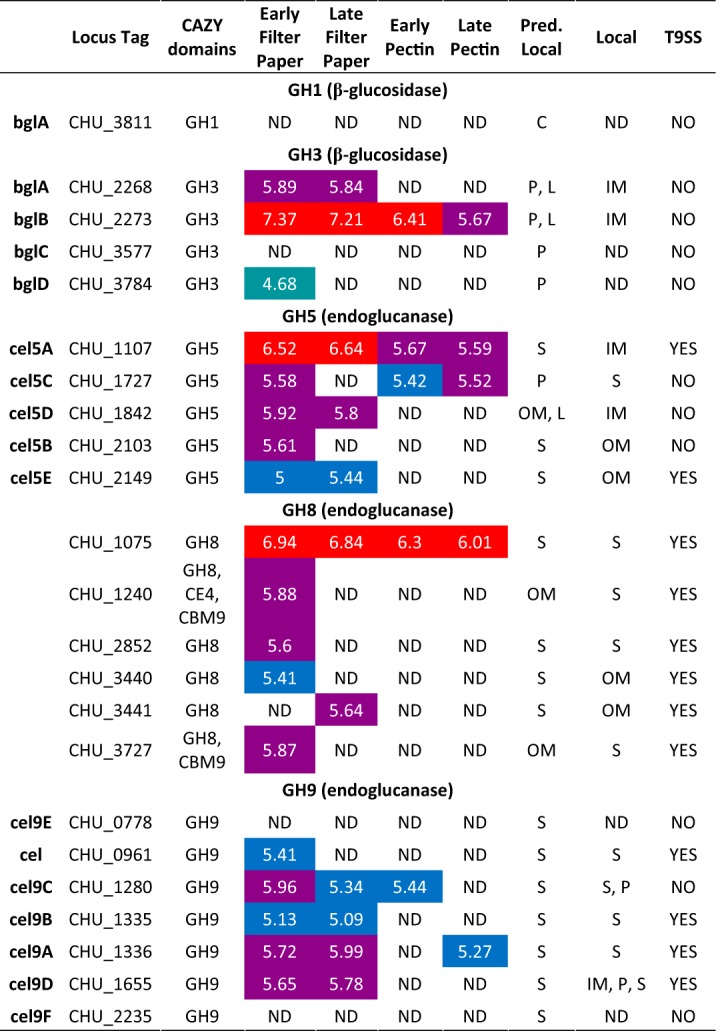
Predicted endoglucanases and β-glucosidases from C. hutchinsonii displaying log_10_ LFQ intensity values during early and late phases of growth with filter paper or pectin as the sole carbon source^*a*^

a The predicted localization is based on prediction using pSORTb 3.0 (65). The proteome localizations were based on overrepresentation in a fraction and are abbreviated as follows: C, cytoplasmic; P, periplasmic; S, secreted extracellular soluble; IM, inner membrane; OM, outer membrane; L, lipoprotein. The presence of the T9SS was based on the presence of the TIGR04183 or TIGR04131 C-terminal domain. Red shading represents log_10_ LFQ intensity levels over 6, purple levels between 5.5 and 5.99, blue levels between 5 and 5.49, and turquoise levels between 4.5 and 4.99. A lack of shading indicates that the levels of the proteins in question were below detectable limits.

Five of the seven GH9 enzymes of C. hutchinsonii were detected in the proteome (Table 1; see also Fig. S1). A double-disruption mutant of the genes encoding the GH9 enzyme CHU_1280 and the GH5 enzyme CHU_2103 (see above) was shown to be unable to digest cellulose (37). CHU_1280 was detected in both the early and late stages of growth on filter paper and in the early stage of growth on pectin (Table 1; see also Fig. S1). Despite previous reports showing a periplasmic localization (37), CHU_1280 was shown in the proteome analysis to be equally abundant in the periplasm and in the extracellular soluble fractions, suggesting that C. hutchinsonii might produce enzyme-loaded outer membrane vesicles (OMV) targeting recalcitrant biomass, similarly to other Gram-negative species such as Fibrobacter succinogenes (49–51). Further experiments are necessary to substantiate this hypothesis. Another GH9 enzyme, CHU_1655, was abundant in both the early and late growth phases on filter paper and was detected in the periplasm and inner membrane as well as in the extracellular soluble fraction. Previous efforts to create a CHU_1655 mutant to obtain a viable strain were unsuccessful (37, 43), which, together with the proteomic data, suggests that CHU_1655 and/or its genetic neighborhood is essential for cellulose digestion or carbohydrate transport (37). The three remaining GH9 enzymes identified in the proteome (CHU_0961, CHU_1335, and CHU_1336) were found in the extracellular soluble fraction during early stages of growth on filter paper. CHU_1335 and CHU_1336 were also present during the later growth stage.

**FIG 2:**
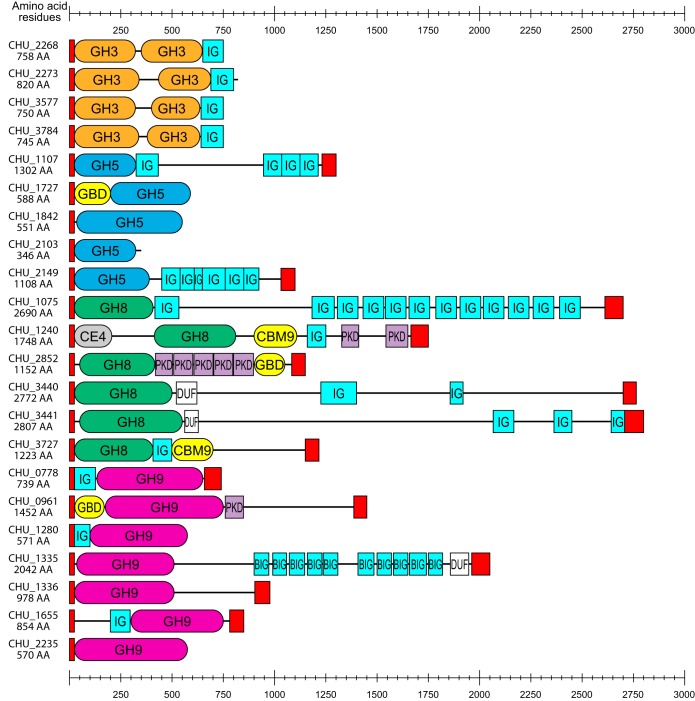
Protein architectures of C. hutchinsonii enzymes putatively involved in cellulose degradation. Red rectangles represent N-terminal signal peptides or C-terminal T9SS sorting domains (TIGR04183 or TIGR04131). Abbreviations: BIG, bacterial Ig-like, group 2 (IPR003343); β-lac, beta-lactamase-related domain (IPR001466); DUF, domain of unknown function; GBD, galactose-binding-like domain; IG, immunoglobulin-like fold; PKD, polycystic kidney disease domain.

GH8 enzymes have curiously been overlooked as potential cellulases in studies of C. hutchinsonii (20, 43), and yet all six encoded putative GH8 enzymes were detected in the proteome. Among those enzymes, CHU_1240 and CHU_3727 contain family 9 carbohydrate binding modules (CBMs), and CHU_1240 also comprises a carbohydrate esterase family 4 (CE4) domain, hinting at a putative role in xylan modification. The remaining GH8 enzymes, CHU_1075, CHU_2852, CHU_3440, and CHU_3441, comprised single GH8 catalytic domains, though they accounted for less than 50% of the total protein lengths (Fig. 2). All GH8 enzymes except for CHU_3441 were detected during early growth on filter paper, though only CHU_1075 and CHU_3441 were detected at the late stages of growth (Table 1; see also Fig. S1). CHU_1075 was abundant also in pectin-grown cells, indicating potential constitutive expression and a key role in carbohydrate turnover. The GH8 domain of CHU_1075 exhibits 32% primary sequence identity with Hungateiclostridium thermocellum (previously Clostridium thermocellum and Ruminiclostridium thermocellum) CelA (Cthe_0269), the primary endoglucanase of H. thermocellum cellulosomes, which suggests endoglucanase activity of the CHU_1075 GH8 domain (3, 52, 53). However, the GH8 domain itself accounts for only ∼400 amino acids in a total protein length of 2,690 amino acids. The remainder of the protein contains a putative galactose-binding domain, several immunoglobulin-like folds, and a CTD (Fig. 2), explaining its abundance in the extracellular soluble fraction (Table 1; see also Fig. S1). The biological role(s) of this and other large GH8-containing proteins is currently unknown.

### β-Glucosidases.

C. hutchinsonii encodes five putative BGs (one GH1 and four GH3s [20]). Three GH3 enzymes (CHU_2268, CHU_2273, and CHU_3784) were detected in the proteome during early stages of growth on filter paper; CHU_2268 and CHU_2273 remained in the late samples and the latter was identified also in pectin-grown cultures (Table 1), which indicates a general role in carbohydrate turnover. While all of the GH3 enzymes were predicted to be periplasmic, the two most abundant members (CHU_2268 and CHU_2273) were abundant also in the inner membrane, and the two enzymes have previously been shown to play redundant key roles in cellobiose utilization (42). The data agree well with both predictions and with previous studies demonstrating localization and activity of BGs in the periplasm without association with the outer membrane (42, 43).

### Hemicellulases and pectinases.

Despite the apparent inability of C. hutchinsonii to metabolize pentoses, several putative hemicellulose-modifying enzymes were detected during the early stages of growth on filter paper (14, 20). These included putative xylan-modifying enzymes (GH10, GH11, CE1, CE3, CE4, CE6, and CE15) and putative xyloglucan-modifying enzymes (GH31 and GH74) (Table S1). However, most of the encoded hemicellulose-modifying enzymes were not present during growth on pectin or during the late stages of growth on filter paper. Thus, hemicellulases are likely produced primarily by cells in direct contact with solid substrates to facilitate access to cellulose fibrils in the plant cell wall, similarly to other cellulose specialist species unable to metabolize pentoses and hemicellulose-derived oligosaccharides (3). Surprisingly, none of the putative pectin/pectate and rhamnogalacturonan lyases (CHU_1162 and CHU_1157, respectively) were detected in the proteome during growth.

### Type 9 secretion system.

The T9SS is essential for crystalline cellulose degradation as it transports proteins containing conserved CTDs across the outer membrane (34). Components of the multiprotein T9SS were detected during growth on both filter paper and pectin (Table S4). Mutating the gene encoding SprP (CHU_0170) has been shown to cause both gliding motility and cellulose utilization defects (34). CHU_0170 was detected throughout growth and was most abundant in the inner membrane. The protein is similar to the Porphyromonas gingivalis PorP, which is believed to form part of the T9SS outer membrane (OM) channel (36, 54). Another putative SprP-like protein, CHU_3434, was found in the outer membrane in both early and late stages of growth on filter paper but was not detected in pectin-grown cultures. The genomic neighborhood of CHU_3434 contains three large proteins without functional annotations (CHU_3435, CHU_3437, and CHU_3439), all detected in the proteome of cells grown on filter paper, in addition to two large GH8 domain-containing proteins (CHU_3440 and CHU_3441; see above). The genomic colocalization and proteomic abundance suggest an important role of the cluster consisting of CHU_3434 to CHU_3441 in the T9SS, gliding motility, and/or cellulose utilization.

10.1128/mSystems.00240-18.7 TABLE S4 Putative genes involved in the type 9 secretion system in C. hutchinsonii displaying the log_10_ LFQ intensity of cells grown on filter paper or pectin as a sole carbon source. The localization was predicted using pSORTb 3.0 (65). The proteome localizations were based on overrepresentation in a fraction and are abbreviated as follows: C, cytoplasmic; P, periplasmic; S, secreted extracellular soluble; IM, inner membrane; OM, outer membrane; L, lipoprotein. Red shading represents log_10_ LFQ intensity levels over 6, purple levels between 5.5 and 5.99, blue levels between 5 and 5.49, and turquoise levels between 4.5 and 4.99. A lack of shading indicates that the levels of the proteins in question were below detectable limits. Download Table S4, DOCX file, 0.02 MB. Copyright © 2018 Taillefer et al. 2018 Taillefer et al. This content is distributed under the terms of the Creative Commons Attribution 4.0 International license.

### Analysis of the S. myxococcoides proteome.

The proteome for S. myxococcoides was obtained and fractionated from cells grown on either filter paper or pectin in a manner similar to that described for C. hutchinsonii, and protein localizations were verified using marker proteins (Table S3). As was seen with C. hutchinsonii, a much greater number of proteins were detected from cells grown on filter paper (1,090 proteins total; 81 CAZymes) than from pectin-grown cells (245 proteins; 13 CAZymes) (see Table S2; see also Fig. S2).

10.1128/mSystems.00240-18.2 FIG S2 Heatmap of the predicted carbohydrate active enzymes from S. myxococcoides detectable during growth on filter paper and/or pectin. Download FIG S2, EPS file, 1.2 MB. Copyright © 2018 Taillefer et al. 2018 Taillefer et al. This content is distributed under the terms of the Creative Commons Attribution 4.0 International license.

### Endoglucanases.

As mentioned above, S. myxococcoides encodes multiple putative EGs (10 putative GH5, 11 GH8, and 8 GH9 enzymes). Among the GH5 enzymes, four (UniProt identifiers MYP_458, MYP_2666, MYP_3206, and MYP_3455) were detected during early stages of growth on filter paper (Table 2; see also Fig. S2). In contrast to C. hutchinsonii, the majority of GH5 members detected in the S. myxococcoides proteome were abundant in the extracellular soluble fraction rather than in the outer membrane and/or periplasm and, furthermore, were detected in both the early and late growth stages on filter paper. Only MYP_3455 was also abundant throughout growth on pectin. MYP_3455 and MYP_458 harbor additional CBM6 domains, which may bind crystalline or amorphous cellulose (Fig. S3) (55). No similar EGs with appended CBM6 domains were found in C. hutchinsonii.

**TABLE 2:**
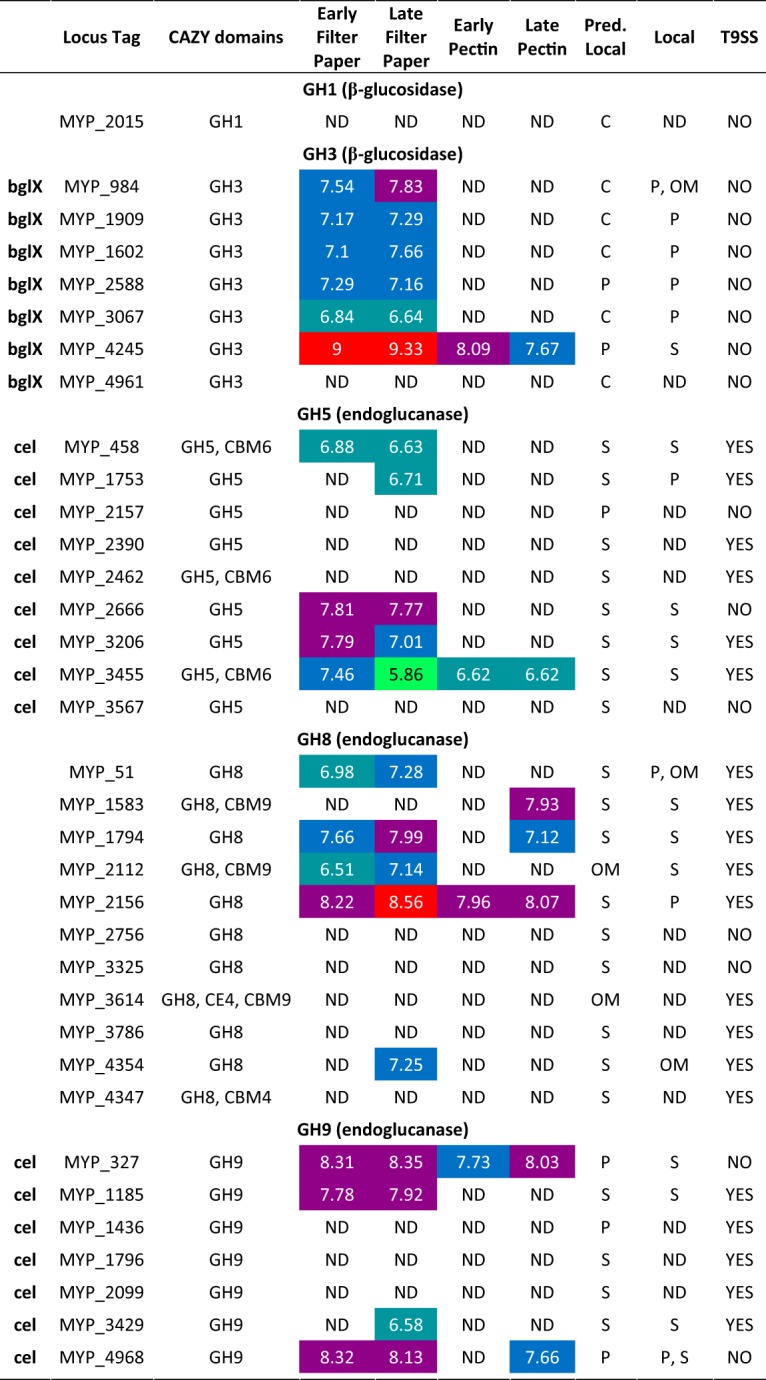
Predicted endoglucanases and β-glucosidases from S. myxococcoides displaying log_10_ LFQ intensity values during early and late phases of growth with filter paper or pectin as the sole carbon source^*a*^

a The predicted localization is based on prediction using pSORTb 3.0 (65). The proteome localizations were based on overrepresentation in a fraction and are abbreviated as follows: C, cytoplasmic; P, periplasmic; S, secreted extracellular soluble; IM, inner membrane; OM, outer membrane; L, lipoprotein. The presence of the T9SS was based on the presence of the TIGR04183 or TIGR04131 C-terminal domain. Red shading represents log_10_ LFQ intensity levels over 8.5, purple levels between 7.75 and 8.49, blue levels between 7 and 7.74, turquoise levels between 6.5 and 6.99, and green levels between 5.5 and 6.49. A lack of shading indicates that the levels of the proteins in question were below detectable limits.

10.1128/mSystems.00240-18.3 FIG S3 Protein architectures of S. myxococcoides enzymes putatively involved in cellulose degradation. Red rectangles represent N-terminal signal peptides or C-terminal T9SS sorting domains (TIGR04183 or TIGR04131). Abbreviations: β-lac, beta-lactamase-related domain (IPR001466); DUF, domain of unknown function; GBD, galactose-binding-like domain; IG, immunoglobulin-like fold; PKD, polycystic kidney disease domain. Download FIG S3, EPS file, 1.2 MB. Copyright © 2018 Taillefer et al. 2018 Taillefer et al. This content is distributed under the terms of the Creative Commons Attribution 4.0 International license.

Six of the putative GH9 enzymes were detected in the proteome, and, similarly to the GH5 enzymes, the majority were most abundant in the extracellular soluble fraction (Table 2; see also Fig. S2). Based on sequence similarity and characterized C. hutchinsonii GH9 enzymes, processive EG activities for certain S. myxococcoides GH9 enzymes can be inferred (48). Four of the GH9 enzymes were detected throughout growth on filter paper; two (MYP_327 and MYP_4968) are putatively processive EGs and one (MYP_1185) a putatively nonprocessive EG. MYP_327 was present in all growth samples, and thus was possibly constitutively expressed, and was abundant in both the extracellular soluble and periplasmic fractions despite lacking a CTD. As with C. hutchinsonii, this points to the possibility of OMVs being utilized also by S. myxococcoides (49–51). MYP_327 shares 61% amino acid identity with CHU_1280, and their similar abundance profiles in the respective proteomes indicate an equivalently important role of MYP_327 in S. myxococcoides.

In contrast to the C. hutchinsonii proteome, the majority of the S. myxococcoides GH8 enzymes were not detected in the proteomic analyses (Table 2; see also Fig. S2). The periplasmic MYP_2156 enzyme was the most abundant GH8 enzyme, detected under all growth conditions. MYP_2156 is not highly similar to any C. hutchinsonii enzyme (36% and 34% amino acid identities with CHU_3440 and CHU_3441, respectively) and differently located compared to the most abundant GH8 enzyme of C. hutchinsonii (CHU_1075; extracellular soluble). Three other GH8 enzymes (MYP_51, MYP_1794, and MYP_2112) were associated mainly with growth on filter paper. Six of the GH8 enzymes (MYP_51, MYP_1794, MYP_2156, MYP_2756, MYP_3325, and MYP_3786) exhibited architectures similar to that of C. hutchinsonii CHU_1075, with very large protein regions of unknown function in addition to the GH8 catalytic domain (Fig. S3).

### β-Glucosidases.

**FIG 3:**
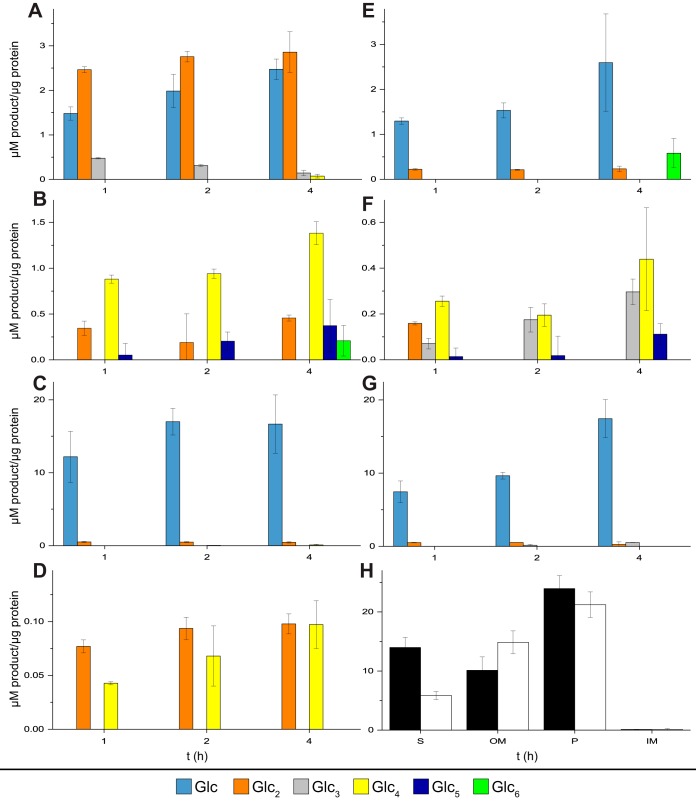
Enzyme activity on cellulose in different protein fractions. Colored bars represent released glucose (light blue), cellobiose (orange), cellotriose (gray), cellotetraose (yellow), cellopentaose (dark blue), and cellohexaose (green) after 1-, 2-, and 4-h reactions. (A to D) Products from cellulose hydrolysis by C. hutchinsonii proteins: extracellular soluble (A), outer membrane (B), periplasm (C), and inner membrane (D). (E to G) Products from cellulose hydrolysis by S. myxococcoides proteins: extracellular soluble (E), outer membrane (F), and periplasmic (G) (no cellulolytic activity was detected in the S. myxococcoides inner membrane fraction). Panel H shows activity on *p*NP-β-glucoside in extracellular soluble (S), outer membrane (OM), periplasmic (P), and inner membrane (IM) fractions from C. hutchinsonii (black) and S. myxococcoides (white) after 1 h of incubation. Error bars represent standard deviations of results from triplicate experiments.

Six putative GH3 β-glucosidases were identified in early and late stages of S. myxococcoides growth on filter paper (Table 2; see also Fig. S2). One, MYP_4245, was also abundant during growth on pectin, indicating possible constitutive expression. Interestingly, this enzyme differed from the C. hutchinsonii β-glucosidases in that it was found in the extracellular soluble fraction, in contrast to its predicted location in the periplasm. The other putative β-glucosidases were all present in the periplasm at abundance levels similar to those seen with the BGs of C. hutchinsonii. S. myxococcoides has previously not been shown to grow on cellobiose without extensive adaptation, which is curious considering the extracellular location of MYP_4245 and the BG activity detected in the secreted soluble fraction (see below and Fig. 3; see also Table S2) (30).

### Hemicellulases, pectinases, and the type 9 secretion system.

In contrast to C. hutchinsonii, the vast majority of putative hemicellulose- and pectin-modifying enzymes of S. myxococcoides were not detected in the proteomes, and these are likely differently regulated in the two species. Putative essential T9SS components of S. myxococcoides were detected under all growth conditions, which indicates constitutive expression of the T9SS (Table S5). While these proteins have not been confirmed to play a role in gliding motility or cellulose turnover, a level of importance of the system in S. myxococcoides similar to that in C. hutchinsonii can be postulated.

10.1128/mSystems.00240-18.8 TABLE S5 Putative genes involved in the type 9 secretion system in S. myxococcoides displaying the log_10_ LFQ intensity of cells grown on filter paper or pectin as a sole carbon source. The localization was predicted using pSORTb 3.0 (65). The proteome localizations were based on overrepresentation in a fraction and are abbreviated as follows: C, cytoplasmic; P, periplasmic; S, secreted extracellular soluble; IM, inner membrane; OM, outer membrane; L, lipoprotein. Red shading represents log_10_ LFQ intensity levels over 8.5, purple levels between 7.75 and 8.49, blue levels between 7 and 7.74, turquoise levels between 6.5 and 6.99, and green levels between 5.5 and 6.49. A lack of coloring indicates that the levels of the proteins in question were below detectable limits. Download Table S5, DOCX file, 0.01 MB. Copyright © 2018 Taillefer et al. 2018 Taillefer et al. This content is distributed under the terms of the Creative Commons Attribution 4.0 International license.

### Enzymatic cellulose hydrolysis.

To potentially correlate the proteomic profiles with enzymatic activity, the different protein fractions from the two species, except cytosolic proteins, were assayed for cellulase and β-glucosidase activities using crystalline cellulose (Avicel) and 4-nitrophenyl-β-glucoside (*p*NP-β-Glc) as substrates (Fig. 3). For cellulose hydrolysis, the activity levels and product profiles differed between both species and cellular fractions. While the extracellular fraction of C. hutchinsonii produced almost equal amounts of glucose and cellobiose, glucose was the main product from S. myxococcoides (Fig. 3A and E). The latter result was unexpected, as S. myxococcoides does not readily grow on glucose, but the activity may be attributable to the main extracellular β-glucosidase, MYP_4245, as mentioned previously, and correlates with the activity on *p*NP-β-Glc in this protein fraction (Fig. 3H). No glucose was detected in reactions using outer membrane proteins (Fig. 3B and F); instead, cellotetraose was the predominant product for C. hutchinsonii and cellotetraose and cellotriose were the predominant products for S. myxococcoides, though longer oligosaccharides were also detected. The reactions performed with periplasmic proteins yielded only glucose (Fig. 3C and G), correlating well with the presence of periplasmic β-glucosidases in both species and with the corresponding highest activities on *p*NP-β-Glc (Fig. 3H). The inner membrane fraction of C. hutchinsonii unexpectedly exhibited cellulase activity (Fig. 3D), which may be attributable to the to-date-uncharacterized inner membrane-located GH5 enzymes CHU_1107 and CHU_1842. S. myxococcoides lacks similarly located corresponding enzymes and consequently exhibited no cellulolytic activity in this cellular compartment.

## DISCUSSION

The cellulose specialist species C. hutchinsonii and S. myxococcoides possess multiple putative cellulose-degrading enzymes, but as neither bacterium encodes known or putative exoglucanases or LPMOs, their mechanisms allowing rapid conversion of cellulose remain enigmatic. Some GH5 and GH9 C. hutchinsonii endoglucanases have been characterized and physiological functions postulated and evaluated through mutational studies (21, 22, 37, 48, 56), but these studies have not been sufficient to explain how crystalline cellulose is converted by this bacterium. Corresponding studies of S. myxococcoides are lacking, despite the likelihood that it uses a highly similar cellulolytic system.

In our proteomic analyses, all GH5 enzymes and most GH9 enzymes from C. hutchinsonii were detected during growth on solid filter paper but only 50% to 60% of the GH5 and GH9 enzymes of S. myxococcoides. Interestingly, C. hutchinsonii GH5 enzyme CHU_2103, which has previously been identified as highly important in cellulose conversion (37, 43, 48), was found only in the early phase of growth on filter paper when the cells were attached to the solid substrate. In contrast, the key GH9 enzyme CHU_1280 was found throughout growth on cellulose. Our data corroborate previous results indicating the importance of these enzymes; further, demonstrating CHU_2103 being located in the outer membrane may explain the strong correlation between cellulase activity and attachment of C. hutchinsonii cells to solid substrates (39).

In the proteomes of both organisms, a large number of GH8 enzymes were abundant during growth on solid filter paper, with CHU_1075 and MYP_2156 being particularly abundant in C. hutchinsonii and S. myxococcoides, respectively. Interestingly, the majority of GH8 enzymes in both species displayed protein architectures where the GH8 domains accounted for relatively small portions of the overall large polypeptides (Fig. 2; see also Fig. S3 in the supplemental material). The main portions of these proteins have no known functions related to cellulose degradation, and their biological role(s) remains speculative. Further, the differences in the cellular locations of the GH8 enzymes in the two bacteria are noteworthy and may point to different roles in cellulose turnover. While all GH8 enzymes of C. hutchinsonii were annotated as extracellular soluble or facing the external environment, the main GH8 enzyme of S. myxococcoides, MYP_2156, was found in the periplasm.

**FIG 4:**
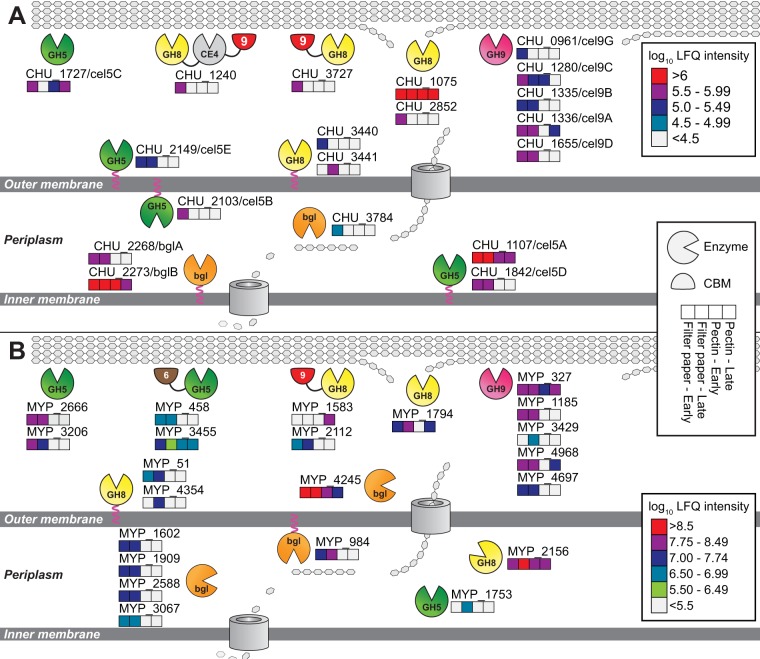
Putative cellulose degradation mechanism in (A) C. hutchinsonii and (B) S. myxococcoides with protein localizations based on abundance in the proteome. Log_10_ LFQ intensity is used to indicate abundance, where numbers below 4.5 and 5.5, respectively, are considered below detectable limits.

The exact mechanisms of cellulose hydrolysis in C. hutchinsonii and S. myxococcoides remain unresolved, but we can infer from our proteomic and enzyme activity data that initial hydrolysis of the cellulose backbone is undertaken by a plethora of putative EGs that are either secreted extracellularly or attached to the outer membrane (Fig. 4). The number of putative EGs located extracellularly in both species creates highly redundant systems which may safeguard against loss of function from single mutation events. Supporting this hypothesis, singular EG disruption mutants in C. hutchinsonii have typically yielded strains without pronounced phenotypes (22, 37, 43, 57). It is highly interesting that additional cellulolytic enzymes are found intracellularly in the periplasm in both species and, for C. hutchinsonii, also in the inner membrane. The role(s) of these intracellular cellulases is puzzling, as transport of cellulose filaments into the periplasm is unlikely, but the bacteria may be able to capture and import longer individual glucan chains for degradation within the periplasm, as has previously been hypothesized (43). The identities of the proteins involved in capture and transport of cellooligosaccharides across the cellular membranes are not known. Possibly, some of the proteins found enriched in the outer membrane fractions, which today lack annotations of functional domains, are important for these processes (see Table S6 in the supplemental material). The majority of proteins of unknown function enriched in the extracellular soluble and outer membrane fractions of both organisms did not contain any previously described features, such as Pfam domains or DUFs (domains of unknown function). Thus, further research is necessary to shed light on their possible biological roles in carbohydrate turnover.

10.1128/mSystems.00240-18.9 TABLE S6 The fifty most abundant proteins of unknown function in the extracellular soluble and outer membrane fractions of C. hutchinsonii and S. myxococcoides, respectively, sorted by log_10_ LFQ intensity. Download Table S6, DOCX file, 0.02 MB. Copyright © 2018 Taillefer et al. 2018 Taillefer et al. This content is distributed under the terms of the Creative Commons Attribution 4.0 International license.

Both C. hutchinsonii and S. myxococcoides have been shown to require direct contact with the substrate for efficient cellulose hydrolysis to occur (14). However, we observed multiple proteins, including putative EGs, being secreted extracellularly in the proteomes in large amounts. During growth, both species produced significant amounts of an extracellular viscous “slime” which has not been structurally characterized to date. Possibly, the secreted proteins may be confined in the extracellular slime matrix in close proximity of the cells, minimizing the amount of solubilized sugars being lost to competing species. Compared to freely secreted enzymes or complexed cellulolytic systems, the semifluid nature of the slime might provide an advantage by allowing penetration of cellulolytic proteins into the crystalline substrate while simultaneously trapping released oligosaccharides. Further investigation is required to evaluate this hypothesis, however.

In summary, by mapping the growth and proteomes of both C. hutchinsonii and S. myxococcoides on various carbohydrates, we provide insight into the carbon source utilization capabilities of both of these cellulose-specialist species and into which proteins are utilized to metabolize crystalline cellulose and soluble pectin—polysaccharides exhibiting different monosaccharide composition, glycosidic linkage, charge, and solubility characteristics. In addition, we pinpoint the localization of different proteins within the cells, which is essential to explain the biology of these two poorly understood cellulolytic species. The plethora of putative cellulose degradation proteins, including many previously unstudied GH8 enzymes, that were abundant throughout the growth process displays the robustness and apparent redundancy of the cellulolytic systems in both C. hutchinsonii and S. myxococcoides. The combined results and the list of proteins of unknown function with putative functions in cellulose turnover (Table S6) provide a number of highly valuable targets for further analyses to identify novel key enzymes for improved cellulose degradation.

## MATERIALS AND METHODS

### Bacterial strains and growth conditions.

C. hutchinsonii G2 (a genome-sequenced glucose-metabolizing strain; referred to as C. hutchinsonii in the text) (20) and S. myxococcoides DSM11118 were grown in McBride minimal medium (MMM) (5.8 mM NaNO_3_, 2 mM MgSO_4_, 6.7 mM KCl, 36 µM FeSO_4_, 0.6 mM NaHCO_3_, 1 mM KH_2_PO_4_; pH 7.3) (58). C. hutchinsonii and S. myxococcoides were grown at 30°C and 25°C, respectively. The MMM was supplemented with 2 g/liter of 0.25-cm^2^ squares of sterile Whatman filter paper or with 2 g/liter of soluble carbohydrates (pectin, xyloglucan, xylan, xylose, mannose, glucose, cellobiose, galactomannan, or glucomannan) as a carbon source. MMM agar was prepared by addition of 6 g/liter agar. Pieces of sterile Whatman filter paper were added on the solidifying agar as the sole carbon source, and the organisms were inoculated in the center of the filter paper strips.

### Protein extraction.

The organisms were grown on MMM-filter paper agar plates for 5 days, and cells were detached from the plates by gentle pipetting of MMM and used to inoculate flasks containing liquid MMM. The cultures were grown for approximately 6 days (early time point) on filter paper until the substrate was coated with yellow cells or for 14 days (late time point) when the medium became yellow due to filter paper disintegration. The cultures were centrifuged at 5,000 × *g* for 15 min, and the resulting supernatant liquids were collected and soluble secreted proteins concentrated using 4 volumes of acetone and −80°C incubation overnight. The cell pellets were resuspended in 15 ml of lysis buffer (1% SDS, 10 mM dithiothreitol [DTT], 100 mM ammonium bicarbonate, pH 8.0) and sonicated for 2 min (3-s pulses, 2-s pauses), followed by centrifugation at 16,000 × *g* for 15 min. The supernatant liquid was precipitated by addition of 4 volumes of acetone and incubation at −80°C overnight followed by centrifugation at 16,000 × *g* for 30 min at 4°C. The pellets were washed with acetone and centrifuged as described above. The acetone was removed, and the protein pellets were resuspended in a mixture containing 200 mM Tris-HCl, 40 mM β-mercaptoethanol, 8% SDS, 0.4% bromophenol blue, and 40% glycerol (pH 6.8), separated by SDS-PAGE using TGX-unstained Mini-Protean gel (Bio-Laboratories, Hercules, CA, USA), and stained using Coomassie brilliant blue R250.

### Cellular fractionation.

The cellular fractions were obtained as previously described (59) from cells grown as described above. At the late time point, the cells were collected by centrifugation at 5,000 × *g* for 15 min. The supernatant liquid was collected as the extracellular soluble fraction. The pelleted cells were washed with 25 ml 30 mM Tris-HCl (pH 8.0) and centrifuged at 5,000 × *g* for 15 min. The cell pellet was resuspended in a reaction mixture containing 25 ml 30 mM Tris-HCl (pH 8.0), 20% sucrose, and 1 mM EDTA and was incubated 10 min at room temperature, followed by centrifugation at 5,000 × *g* for 15 min. The pellet was resuspended in 25 ml of ice-cold 5 mM MgSO_4_, incubated on ice for 10 min, and centrifuged at 5,000 × *g* for 20 min. The supernatant liquid was collected as the periplasmic fraction. The resulting pellet was resuspended in 20 ml of 50 mM Tris-HCl (pH 8.0) and lysed by sonication followed by centrifugation at 16,000 × *g* for 30 min. The supernatant liquid was centrifuged at 100,000 × *g* for 1 h at 4°C. Resulting supernatant liquid was collected as the cytoplasmic fraction. The pellet containing the total membranes was dissolved in a reaction mixture containing 50 mM Tris-HCl (pH 7.5), 10 mM MgCl_2_, and 2% Triton X-100 and incubated at room temperature with shaking for 1 h, followed by centrifugation at 100,000 × *g* for 1 h at 4°C. The supernatant liquid was collected as the inner membrane fraction and the pellet as the outer membrane fraction. Outer membrane protein A (ompA; CHU_1710 and MYP_505), periplasmic phosphate binding protein (pstS; CHU_3818 and MYP_603), and NADH dehydrogenase subunit L (nuoL; CHU_1371 and MYP_729) were used as markers to assess the fractionation of the outer membrane, periplasm, and inner membrane, respectively (see Table S3 in the supplemental material).

### In-gel trypsinization and mass-spectrometric and bioinformatic analyses.

Peptides were prepared as described by Arntzen et al. (60) and subjected to in-gel trypsinization as described by Shevchenko et al. (61). The dried peptides were analyzed by the use of NanoLC-Orbitrap tandem mass spectrometry (MS/MS) as described previously (58). MS raw files were analyzed using MaxQuant v1.5.8.3 (62) for label-free quantification (LFQ) of proteins and the MaxQuant label-free algorithm (47) with both unique and razor peptides for protein quantification. Protein identification was done using the Andromeda database search engine (63) for searches against the UniProt proteome set for C. hutchinsonii (UP000001822; 3,771 sequences) and S. myxococcoides (UP000030185; 5,042 sequences). Identifications were filtered to achieve a protein false-discovery rate (FDR) of 1%. Proteins identified in two of the three biological replicates were considered valid. Overrepresentation of the proteins in the fractions was calculated as a percentage of the protein LFQ intensity value corresponding to the sum of all LFQ intensities in the fraction.

### Enzyme assays.

The cellular fractions were separated and collected as previously described. The outer membrane fraction was resuspended in 50 mM Tris-HCl (pH 8.0). Proteins were concentrated by centrifugation performed with Pierce protein concentrators (Thermo Scientific, Bremen, Germany) (3 K molecular weight cutoff [MWCO]). Avicel degradation activity was determined in 200-µl reaction mixtures containing 2 g/liter Avicel PH-101 (Sigma-Aldrich, Stockholm, Sweden), 50 mM sodium citrate (pH 7.0), 5 mM CaCl_2_, and 10 µg protein (determined by the Bradford assay). The assays were incubated with shaking in round-bottomed tubes at 30°C and were stopped by the addition of 50 µl 5 M NaOH. Products were determined using a Dionex ICS-5000 system equipped with a CarboPac PA-200 analytical column (Dionex, Sunnyvale, CA, USA) (3 by 250 mm) and a flow rate of 0.5 ml/min. Eluent A consisted of water, eluent B of 300 mM NaOH, and eluent C of 1 M sodium acetate. The method used was as follows: 0 to 5 min, 15% eluent B; 5 to 15 min, a linear gradient of 15% to 33% eluent B; 15 to 25 min, 33% eluent B and a linear gradient of 0% to 10% C; 25 to 30 min, 33% eluent B and 66% eluent C. Products were detected by pulsed amperometric detection (PAD) using Chromeleon software v.7 (Thermo Scientific, Bremen, Germany). β-Glucosidase activity was assayed using *p*NP-β-Glc in 96-well plates and 200-μl reaction mixtures containing 25 mM sodium citrate (pH 7.0), 5 mM CaCl_2_, 10 mM *p*NP-β-Glc, and 1 to 10 μg protein, and the plates were incubated at 30°C for 1 h. The reactions were stopped by addition of 100 μl 0.4 M glycine (pH 10.8), and the products were quantified at 405 nm using a FluoStar Omega plate reader (BMG Labtech, Ortenberg, Germany) and a *p*-nitrophenol standard curve.

### Data availability.

The proteomics data have been deposited in the ProteomeXchange consortium (http://proteomecentral.proteomexchange.org) via the PRIDE partner repository (64) with the data set identifier PXD010836.
