# Supplementary material for: Proteomic Dissection of the Cellulolytic Machineries Used by Soil-Dwelling Bacteroidetes
Source: mSystems. 2018 Nov 20;3(6):e00240-18. doi: 10.1128/mSystems.00240-18 (PMC6247017; doi:10.1128/mSystems.00240-18)
Supplement: TABLE S1 [file sys006182297st1.docx]

**Table S1**

| **Name** | **Locus Tag** | **CAZY domains** | **Early Filter Paper** | **Late Filter Paper** | **Early Pectin** | **Late Pectin** | **Pred. Local** | **Local** | **T9SS** |
| --- | --- | --- | --- | --- | --- | --- | --- | --- | --- |
| **GH1 (β-glucosidase)** | | | | | | | | | |
| **bglA** | CHU_3811 | GH1 | ND | ND | ND | ND | C | ND | NO |
| **GH3 (β-glucosidase)** | | | | | | | | | |
| **nagA** | CHU_0013 | GH3 | 4.75 | ND | ND | ND | IM | ND | NO |
| **nagZ** | CHU_0470 | GH3 | 5.49 | 6.036 | ND | ND | C | ND | NO |
| **bglX** | CHU_2268 | GH3 | 5.89 | 5.84 | ND | ND | P, L | IM | NO |
| **bglX** | CHU_2273 | GH3 | 7.37 | 7.21 | 6.41 | 5.67 | P, L | IM | NO |
| **bglX** | CHU_3577 | GH3 | ND | ND | ND | ND | P | ND | NO |
| **bglX** | CHU_3784 | GH3 | 4.68 | ND | ND | ND | P | ND | NO |
| **GH5 (endoglucanase)** | | | | | | | | | |
| **cel** | CHU_1107 | GH5_2 | 6.52 | 6.64 | 5.67 | 5.59 | S | IM | YES |
| **cel** | CHU_1727 | GH5 | 5.58 | ND | 5.42 | 5.52 | P | S | NO |
| **cel** | CHU_1842 | GH5 | 5.92 | 5.80 | ND | ND | OM, L | IM | NO |
| **cel** | CHU_2103 | GH5_2 | 5.61 | ND | ND | ND | S | OM | NO |
| **cel** | CHU_2149 | GH5 | 5.00 | 5.44 | ND | ND | S | OM | YES |
| **GH8 (endoglucanase)** | | | | | | | | | |
|  | CHU_1075 | GH8 | 6.94 | 6.84 | 6.30 | 6.01 | S | S | YES |
|  | CHU_1240 | GH8, CE4, CBM9 | 5.88 | ND | ND | ND | OM | S | YES |
|  | CHU_2852 | GH8 | 5.60 | ND | ND | ND | S | S | YES |
|  | CHU_3440 | GH8 | 5.41 | ND | ND | ND | S | OM | YES |
|  | CHU_3441 | GH8 | ND | 5.64 | ND | ND | S | OM | YES |
|  | CHU_3727 | GH8, CBM9 | 5.87 | ND | ND | ND | OM | S | YES |
| **GH9 (endoglucanase)** | | | | | | | | | |
| **cel** | CHU_0778 | GH9 | ND | ND | ND | ND | S | ND | NO |
| **cel** | CHU_0961 | GH9 | 5.41 | ND | ND | ND | S | S | YES |
| **cel** | CHU_1280 | GH9 | 5.96 | 5.34 | 5.44 | ND | S | S, P | NO |
| **cel** | CHU_1335 | GH9 | 5.13 | 5.09 | ND | ND | S | S | YES |
| **cel** | CHU_1336 | GH9 | 5.72 | 5.99 | ND | 5.27 | S | S | YES |
| **cel** | CHU_1655 | GH9 | 5.65 | 5.78 | ND | ND | S | IM, P, S | YES |
| **cel** | CHU_2235 | GH9 | ND | ND | ND | ND | S | ND | NO |
| **GH10 (xylanase)** | | | | | | | | | |
|  | CHU_1239 | GH10, CE6, CBM4 | 4.69 | ND | ND | ND | S | ND | YES |
|  | CHU_2043 | GH10, CBM9 | 5.10 | ND | ND | ND | S | ND | YES |
| **xynT** | CHU_2105 | GH10, CBM4 | ND | ND | ND | ND | P | ND | YES |
| **GH11 (xylanase)** | | | | | | | | | |
|  | CHU_2379 | GH11, CBM9 | 5.81 | ND | ND | ND | OM | ND | YES |
| **GH13 (amylase)** | | | | | | | | | |
| **amyA** | CHU_0959 | GH13 | ND | ND | ND | ND | C | ND |  |
| **glgB** | CHU_1345 | GH13_8, CBM48 | 6.49 | 5.94 | 5.35 | 5.49 | C | P | NO |
| **amyA** | CHU_2409 | GH13 | 5.74 | 6.06 | ND | ND | C | ND | NO |
| **treC** | CHU_2602 | GH13_26 | 4.62 | ND | ND | ND | C | ND | NO |
| **GH15 (glucoamylase)** | | | | | | | | | |
|  | CHU_0399 | GH15 | ND | ND | ND | ND | C | ND | NO |
| **GH16 (β-1,3/1,4-glucanase, licheninase, xyloglucanase)** | | | | | | | | | |
|  | CHU_0981 | GH16 | ND | ND | ND | ND | S | ND | NO |
| **bglC** | CHU_2802 | GH16 | ND | ND | ND | ND | S | ND | NO |
| **CH23 (peptidoglycan lytic transglycosylase)** | | | | | | | | | |
| **mltD** | CHU_0026 | GH23, CBM50 | ND | ND | ND | ND | OM | ND | NO |
| **mltD** | CHU_0157 | GH23 | ND | ND | ND | ND | IM | ND | NO |
|  | CHU_2792 | GH23 | ND | ND | ND | ND | C | ND | NO |
| **mltD** | CHU_3030 | GH23, CBM50 | ND | ND | ND | ND | P, L | ND | NO |
| **GH26 (β-1,4-mannanase)** | | | | | | | | | |
|  | CHU_0353 | GH26, CBM35 | 4.84 | ND | ND | ND | S | IM | YES |
| **GH30 (xylanase, xylosidase)** | | | | | | | | | |
|  | CHU_2042 | GH30_8, CBM9 | ND | ND | ND | ND | OM | ND | YES |
| **GH31 (α-glucosidase, α-xylosidase)** | | | | | | | | | |
| **malZ** | CHU_0803 | GH31 | 5.25 | 4.84 | ND | ND | C | C | NO |
| **GH43 (β-xylosidase, α-l-arabinofuranosidase)** | | | | | | | | | |
|  | CHU_2041 | GH43, CE6, CBM9 | ND | ND | ND | ND | OM | ND | YES |
|  | CHU_2044 | GH43, CBM6, CBM9 | ND | ND | ND | ND | OM | ND | YES |
| **GH57 (amylase)** | | | | | | | | | |
|  | CHU_0801 | GH57 | 5.09 | ND | ND | ND | C | ND | NO |
| **GH73 (peptidoglycan hydrolase)** | | | | | | | | | |
| **flgJ** | CHU_1472 | GH73 | ND | ND | ND | ND | P | ND | NO |
| **GH74 (xyloglucanase)** | | | | | | | | | |
|  | CHU_1155 | GH74 | ND | ND | ND | ND | S | ND | YES |
| **GH77 (4-α-glucanotransferase)** | | | | | | | | | |
| **malQ** | CHU_2290 | GH77 | 6.05 | 5.54 | ND | ND | C | OM | NO |
| **GH133 (glycogen debranching enzyme)** | | | | | | | | | |
|  | CHU_3810 | GH133 | 6.26 | 6.14 | 5.45 | 5.40 | C | C | NO |
| **CE1 (acetyl-xylan esterase, feruloyl esterase)** | | | | | | | | | |
|  | CHU_2040 | CE1, CBM9 | ND | ND | ND | ND | OM | ND | YES |
|  | CHU_2408 | CE1 | ND | ND | ND | ND | C | ND | NO |
| **CE2 (acetyl-xylan esterase)** | | | | | | | | | |
| **gatC** | CHU_0721 | CE2 | ND | ND | ND | ND | OM, L | ND | NO |
|  | CHU_3337 | CE2 | ND | ND | ND | ND | OM, L | ND | NO |
| **CE4 (acetyl-xylan esterase, chitin deacetylase)** | | | | | | | | | |
|  | CHU_0603 | CE4 | ND | ND | ND | ND | C | ND | NO |
|  | CHU_1240 | CE4, GH8, CBM9 | 5.88 | ND | ND | ND | OM | S | YES |
| **yheN** | CHU_2113 | CE4 | ND | ND | ND | ND | C | ND | NO |
|  | CHU_2175 | CE4 | ND | ND | ND | ND | C | ND | NO |
| **yxkH** | CHU_3339 | CE4 | ND | ND | ND | ND | S | ND | YES |
| **CE6 (acetyl-xylan esterase)** | | | | | | | | | |
|  | CHU_1239 | CE6, GH10, CBM4 | 4.69 | ND | ND | ND | S | ND | YES |
|  | CHU_2041 | GH43, CE6, CBM9 | ND | ND | ND | ND | OM | ND | YES |
| **CE11 (UDP-3-O-acyl N-acetylglucosamine deacetylase)** | | | | | | | | | |
| **lpxC fabZ** | CHU_1037 | CE11 | 6.23 | 6.41 | 5.64 | ND | C | OM | NO |
| **CE14 (N-acetyl-1-d-myo-inosityl-2-amino-2-deoxy-α-d-glucopyranoside deacetylase)** | | | | | | | | | |
|  | CHU_1776 | CE14 | 4.94 | ND | ND | ND | C | C | NO |
| **CE15 (glucuronoyl esterase)** | | | | | | | | | |
|  | CHU_1238 | CE15, CBM9 | 5.56 | ND | ND | ND | OM | ND | YES |
| **CBM4 (xylan, β-1,3-glucan, β-1,3/1,4-glucan, β-1,6-glucan and amorphous cellulose)** | | | | | | | | | |
|  | CHU_1239 | CE6, GH10, CBM4 | 4.69 | ND | ND | ND | S | ND | YES |
| **xynT** | CHU_2105 | GH10, CBM4 | ND | ND | ND | ND | P | ND | YES |
|  | CHU_2044 | GH43, CBM4, CBM9 | ND | ND | ND | ND | OM | ND | NO |
| **CBM6 (xylan)** | | | | | | | | | |
|  | CHU_1051 | CBM6, CBM51 | ND | ND | ND | ND | S | ND | NO |
| **CBM35 (xylan)** | | | | | | | | | |
|  | CHU_0353 | GH26, CBM35 | 4.84 | ND | ND | ND | S | IM | NO |
| **CBM50 (peptidoglycan)** | | | | | | | | | |
| **mltD** | CHU_0026 | GH23, CBM50 | ND | ND | ND | ND | OM | ND | NO |
|  | CHU_1777 | CBM50 | ND | ND | ND | ND | OM | ND | NO |
|  | CHU_2842 | CBM50 | 5.76 | 5.47 | ND | ND | S | ND | NO |
| **mltD** | CHU_2994 | CBM50 | 5.40 | ND | ND | ND | S | ND | NO |
| **mltD** | CHU_3030 | GH23, CBM50 | ND | ND | ND | ND | P, L | ND | NO |
| **CBM62 (galactose)** | | | | | | | | | |
|  | CHU_2399 | CBM62 | ND | ND | ND | ND | S | ND | NO |
| **PL1 (pectin lyase)** | | | | | | | | | |
|  | CHU_1162 | PL1 | ND | ND | ND | ND | S | ND | NO |
| **PL11 (rhamnogalacturonan lyase)** | | | | | | | | | |
|  | CHU_1157 | PL11 | ND | ND | ND | ND | S | ND | NO |
| **PL14 (alginate lyase)** | | | | | | | | | |
|  | CHU_2148 | PL14_3 | ND | ND | ND | ND | S | ND | NO |
| **GT2 (β-glycans)** | | | | | | | | | |
|  | CHU_0121 | GT2 | 5.93 | 5.81 | 6.11 | 6.14 | C | OM | NO |
| **lgtF** | CHU_0605 | GT2 | ND | ND | ND | ND | C | ND | NO |
|  | CHU_0852 | GT2 | ND | ND | ND | ND | C | ND | NO |
|  | CHU_0853 | GT2 | ND | ND | ND | ND | C | ND | NO |
|  | CHU_0858 | GT2 | ND | ND | ND | ND | C | ND | NO |
|  | CHU_0860 | GT2 | ND | ND | ND | ND | C | ND | NO |
|  | CHU_0863 | GT2 | 4.87 | ND | ND | ND | C | ND | NO |
|  | CHU_0884 | GT2 | ND | ND | ND | ND | C | ND | NO |
|  | CHU_0888 | GT2 | 4.70 | ND | ND | ND | IM | ND | NO |
|  | CHU_0896 | GT2 | 4.76 | ND | ND | ND | C | IM | NO |
| **ycdQ** | CHU_0912 | GT2 | ND | ND | ND | ND | OM | ND | NO |
|  | CHU_1044 | GT2 | ND | ND | ND | ND | IM | ND | NO |
|  | CHU_1196 | GT2 | ND | ND | ND | ND | IM | ND | NO |
| **lgtD** | CHU_1573 | GT2 | 4.98 | ND | ND | ND | C | ND | NO |
|  | CHU_1791 | GT2 | ND | ND | ND | ND | IM | ND | NO |
|  | CHU_1792 | GT2 | ND | ND | ND | ND | IM | ND | NO |
|  | CHU_2109 | GT2 | ND | ND | ND | ND | IM | ND | NO |
| **ycdQ** | CHU_2174 | GT2 | ND | ND | ND | ND | IM | ND | NO |
| **ycdQ** | CHU_2182 | GT2 | ND | ND | ND | ND | IM | ND | NO |
|  | CHU_2506 | GT2 | ND | ND | ND | ND | IM | ND | NO |
|  | CHU_2532 | GT2 | ND | ND | ND | ND | C | ND | NO |
| **yfdH** | CHU_2681 | GT2 | ND | ND | ND | ND | IM | ND | NO |
| **wcaE** | CHU_2772 | GT2 | ND | ND | ND | ND | IM | ND | NO |
|  | CHU_2999 | GT2 | ND | ND | ND | ND | IM | ND | NO |
|  | CHU_3003 | GT2 | 5.64 | 6.57 | 5.86 | ND | IM | IM | NO |
|  | CHU_3457 | GT2 | ND | ND | ND | ND | C | ND | NO |
|  | CHU_3770 | GT2 | ND | ND | ND | ND | IM | ND | NO |
| **GT3 (glycogen synthase)** | | | | | | | | | |
| **glgA** | CHU_1581 | GT3 | 6.60 | 6.16 | 5.76 | 5.51 | C | OM | NO |
| **GT4 (α-glycans)** | | | | | | | | | |
|  | CHU_0012 | GT4 | 6.22 | 5.80 | ND | ND | C | OM | NO |
|  | CHU_0061 | GT4 | ND | ND | ND | ND | C | ND | NO |
|  | CHU_0201 | GT4 | ND | ND | ND | ND | C | ND | NO |
| **wcaL** | CHU_0604 | GT4 | ND | ND | ND | ND | C | ND | NO |
|  | CHU_0802 | GT4, GT5 | 5.37 | 5.44 | ND | ND | C | ND | NO |
|  | CHU_0851 | GT4 | ND | ND | ND | ND | C | ND | NO |
|  | CHU_0857 | GT4 | ND | ND | ND | ND | C | ND | NO |
| **wcaL** | CHU_0868 | GT4 | ND | ND | ND | ND | C | ND | NO |
|  | CHU_0869 | GT4 | ND | ND | ND | ND | C | ND | NO |
| **wcaL** | CHU_0870 | GT4 | ND | ND | ND | ND | C | ND | NO |
|  | CHU_0885 | GT4 | ND | ND | ND | ND | IM | ND | NO |
|  | CHU_0889 | GT4 | 5.49 | 5.75 | ND | ND | C | OM | NO |
|  | CHU_0890 | GT4 | 5.75 | 5.81 | ND | ND | C | OM | NO |
|  | CHU_0894 | GT4 | ND | ND | ND | ND | C | ND | NO |
|  | CHU_0895 | GT4 | 5.20 | ND | ND | ND | C | ND | NO |
| **wca** | CHU_0913 | GT4 | 4.98 | ND | ND | ND | C | ND | NO |
| **rfaG** | CHU_0928 | GT4 | 5.90 | 5.92 | ND | ND | C | P, OM | NO |
|  | CHU_0930 | GT4 | ND | ND | ND | ND | C | ND | NO |
|  | CHU_2781 | GT4 | ND | ND | ND | ND | C | ND | NO |
| **wcaL** | CHU_2884 | GT4 | ND | ND | ND | ND | C | ND | NO |
| **hemG** | CHU_2890 | GT4 | ND | ND | ND | ND | C | ND | NO |
|  | CHU_2891 | GT4 | ND | ND | ND | ND | OM | ND | NO |
| **wcaL** | CHU_2892 | GT4 | ND | ND | ND | ND | C | ND | NO |
|  | CHU_3692 | GT4 | ND | ND | ND | ND | C | ND | NO |
|  | CHU_3842 | GT4 | ND | ND | ND | ND | C | ND | NO |
| **GT5 (glycogen synthase)** | | | | | | | | | |
| **glgA** | CHU_3836 | GT5 | 5.20 | 5.34 | ND | ND | C | C | NO |
| **GT9 (lipopolysaccharide N-acetylglucosaminyltransferase)** | | | | | | | | | |
| **rfaQ** | CHU_1683 | GT9 | ND | ND | ND | ND | C | ND | NO |
| **GT14 (β-1,3-galactosyl-O-glycosyl-glycoprotein β-1,6-N-acetylglucosaminyltransferase)** | | | | | | | | | |
|  | CHU_0861 | GT14 | ND | ND | ND | ND | C | ND | NO |
| **GT19 (lipid-A-disaccharide synthase)** | | | | | | | | | |
| **nuoB2** | CHU_1381 | GT19 | 6.31 | 5.46 | ND | ND | C | OM | NO |
| **GT20 (trehalose-6-P phosphatase)** | | | | | | | | | |
| **otsA** | CHU_0400 | GT20 | 6.03 | 5.53 | ND | ND | C | C | NO |
| **GT28 (1,2-diacylglycerol 3-β-galactosyltransferase)** | | | | | | | | | |
| **murG** | CHU_2739 | GT28 | 5.67 | 5.92 | ND | ND | IM | C | NO |
|  | CHU_3812 | GT28 | ND | ND | ND | ND | C | ND | NO |
| **GT30 (CMP-β-KDO: α-3-deoxy-d-manno-octulosonic-acid (KDO) transferase)** | | | | | | | | | |
| **kdtA** | CHU_2726 | GT30 | ND | ND | ND | ND | C | ND | NO |
| **GT35 (glycogen or starch phosphorylase)** | | | | | | | | | |
| **glgP** | CHU_0308 | GT35 | 5.13 | ND | ND | ND | C | OM | NO |
| **GT51 (murein polymerase)** | | | | | | | | | |
|  | CHU_0176 | GT51 | 5.13 | 5.39 | ND | ND | IM | IM | NO |
| **pbpC** | CHU_1192 | GT51 | ND | ND | ND | ND | IM | ND | NO |
|  | CHU_1251 | GT51 | ND | ND | ND | ND | IM | ND | NO |
| **mtgA** | CHU_1314 | GT51 | ND | ND | ND | ND | IM | ND | NO |
| **mrcA** | CHU_1888 | GT51 | 6.14 | 5.77 | ND | ND | IM | IM, P | NO |
| **ponA** | CHU_3131 | GT51 | 4.45 | ND | ND | ND | IM | ND | NO |
| **GT83 (undecaprenyl phosphate-α-l-Ara4N: 4-amino-4-deoxy-β-l-arabinosyltransferase)** | | | | | | | | | |
| **arnT** | CHU_3370 | GT83 | ND | ND | ND | ND | IM | ND | NO |
| **arnT** | CHU_3773 | GT83 | ND | ND | ND | ND | IM | ND | NO |
