# Supplementary material for: Proteomic Dissection of the Cellulolytic Machineries Used by Soil-Dwelling Bacteroidetes
Source: mSystems. 2018 Nov 20;3(6):e00240-18. doi: 10.1128/mSystems.00240-18 (PMC6247017; doi:10.1128/mSystems.00240-18)
Supplement: TABLE S2 [file sys006182297st2.docx]

**Table S2**

|  | **Locus Tag** | **CAZY domains** | **Early Filter Paper** | **Late Filter Paper** | **Early Pectin** | **Late Pectin** | **Pred. Local** | **Local** | **T9SS** |
| --- | --- | --- | --- | --- | --- | --- | --- | --- | --- |
| **GH1 (β-glucosidase)** | | | | | | | | | |
|  | MYP_2015 | GH1 | ND | ND | ND | ND | C | ND | NO |
| **GH2 (β-galactosidase)** | | | | | | | | | |
|  | MYP_3616 | GH2 | 7.05 | 7.05 | ND | ND | OM | P | NO |
| **GH3 (β-glucosidase)** | | | | | | | | | |
|  | MYP_984 | GH3 | 7.54 | 7.83 | ND | ND | C | P, OM | NO |
|  | MYP_1909 | GH3 | 7.17 | 7.29 | ND | ND | C | P | NO |
|  | MYP_1602 | GH3 | 7.10 | 7.66 | ND | ND | C | P | NO |
|  | MYP_2588 | GH3 | 7.29 | 7.16 | ND | ND | P | P | NO |
|  | MYP_3067 | GH3 | 6.84 | 6.64 | ND | ND | C | P | NO |
|  | MYP_4245 | GH3 | 9.00 | 9.33 | 8.09 | 7.67 | P | S | NO |
|  | MYP_4961 | GH3 | ND | ND | ND | ND | C | ND | NO |
| **GH5 (endoglucanase)** | | | | | | | | | |
|  | MYP_458 | GH5, CBM6 | 6.88 | 6.63 | ND | ND | S | S | YES |
|  | MYP_3206 | GH5 | 7.79 | 7.01 | ND | ND | S | S | YES |
|  | MYP_2390 | GH5_2 | ND | ND | ND | ND | S | ND | YES |
|  | MYP_2666 | GH5_2 | 7.81 | 7.77 | ND | ND | S | S | NO |
|  | MYP_2462 | GH5_2, CBM6 | ND | ND | ND | ND | S | ND | YES |
|  | MYP_3455 | GH5_2, CBM6 | 7.46 | 5.86 | 6.62 | 6.62 | S | S | YES |
|  | MYP_3567 | GH5_2 | ND | ND | ND | ND | S | ND | NO |
|  | MYP_2157 | GH5_7 | ND | ND | ND | ND | P | ND | NO |
|  | MYP_1753 | GH5 | ND | 6.71 | ND | ND | S | P | YES |
| **GH8 (endoglucanase)** | | | | | | | | | |
|  | MYP_51 | GH8 | 6.98 | 7.28 | ND | ND | S | P, OM | YES |
|  | MYP_1583 | GH8, CBM9 | ND | ND | ND | 7.93 | S | S | YES |
|  | MYP_1794 | GH8 | 7.66 | 7.99 | ND | 7.12 | S | S | YES |
|  | MYP_2112 | GH8, CBM9 | 6.51 | 7.14 | ND | ND | OM | S | YES |
|  | MYP_2156 | GH8 | 8.22 | 8.56 | 7.96 | 8.07 | S | P | YES |
|  | MYP_2756 | GH8 | ND | ND | ND | ND | S | ND | NO |
|  | MYP_3325 | GH8 | ND | ND | ND | ND | S | ND | NO |
|  | MYP_3614 | GH8, CE4, CBM9 | ND | ND | ND | ND | OM | ND | YES |
|  | MYP_3786 | GH8 | ND | ND | ND | ND | S | ND | YES |
|  | MYP_4354 | GH8 | ND | 7.25 | ND | ND | S | OM | YES |
|  | MYP_4347 | GH8, CBM4 | ND | ND | ND | ND | S | ND | YES |
| **GH9 (endoglucanase)** | | | | | | | | | |
|  | MYP_327 | GH9 | 8.31 | 8.35 | 7.73 | 8.03 | P | S | NO |
|  | MYP_1185 | GH9 | 7.78 | 7.92 | ND | ND | S | S | YES |
|  | MYP_1436 | GH9 | ND | ND | ND | ND | P | ND | YES |
|  | MYP_2099 | GH9 | ND | ND | ND | ND | S | ND | YES |
|  | MYP_1796 | GH9 | ND | ND | ND | ND | S | ND | YES |
|  | MYP_3429 | GH9 | ND | 6.58 | ND | ND | S | S | YES |
|  | MYP_4967 | GH9 | 7.13 | 7.51 | ND | ND | P | OM, S | NO |
|  | MYP_4968 | GH9 | 8.32 | 8.13 | ND | 7.66 | P | P, S | NO |
| **GH10 (xylanase)** | | | | | | | | | |
|  | MYP_2074 | GH10, CBM4 | ND | ND | ND | ND | S | ND | YES |
|  | MYP_2104 | GH10, CBM6 | ND | ND | ND | ND | S | ND | YES |
|  | MYP_3331 | GH10, CBM6 | ND | ND | ND | ND | S | ND | YES |
| **GH11 (xylanase)** | | | | | | | | | |
|  | MYP_2111 | GH11, CBM9 | ND | ND | ND | ND | S | ND | YES |
| **GH13 (amylase)** | | | | | | | | | |
|  | MYP_2299 | GH13 | ND | ND | ND | ND | C | ND | NO |
|  | MYP_4685 | GH13_8 | 7.35 | 7.83 | 7.38 | ND | C | P | NO |
|  | MYP_3208 | GH13_42 | ND | ND | ND | ND | S | ND | NO |
| **GH15 (glucoamylase)** | | | | | | | | | |
|  | MYP_3689 | GH15 | 7.10 | 7.92 | ND | ND | C | C | NO |
| **GH16 (β-1,3/1,4-glucanase, licheninase, xyloglucanase)** | | | | | | | | | |
|  | MYP_2076 | GH16 | ND | ND | ND | ND | S | ND | NO |
|  | MYP_3454 | GH16, CBM16 | ND | ND | ND | ND | S | ND | YES |
|  | MYP_4151 | GH16 | ND | ND | ND | ND | S | ND | NO |
|  | MYP_4349 | GH16, CBM4 | ND | 6.91 | ND | ND | S | S | YES |
| **GH18 (chitinase)** | | | | | | | | | |
|  | MYP_3548 | GH18 | 6.56 | 6.99 | ND | ND | IM | P | NO |
| **GH23 (peptidoglycan lytic transglycosylase)** | | | | | | | | | |
|  | MYP_105 | GH23, CBM50 | ND | ND | ND | ND | C | ND | NO |
|  | MYP_913 | GH23 | ND | ND | ND | ND | U | ND | NO |
|  | MYP_971 | GH23, CBM50 | ND | ND | ND | ND | C | ND | NO |
|  | MYP_1703 | GH23 | ND | ND | ND | ND | C | ND | NO |
|  | MYP_4307 | GH23, CBM50 | ND | ND | ND | ND | S | ND | NO |
| **GH26 (β-1,4-mannanase)** | | | | | | | | | |
|  | MYP_4610 | GH26, CBM35 | ND | 7.17 | ND | ND | U | ND | YES |
| **GH30 (xylanase, xylosidase)** | | | | | | | | | |
|  | MYP_2082 | GH30_8, CBM6 | ND | ND | ND | ND | S | ND | YES |
| **GH31 (α-glucosidase, xylosidase)** | | | | | | | | | |
|  | MYP_331 | GH31 | 6.96 | 8.20 | ND | 7.41 | C | C | NO |
| **GH37 (α,α-trehalase)** | | | | | | | | | |
|  | MYP_581 | GH37 | ND | ND | ND | ND | C | ND | NO |
| **GH43 (β-xylosidase, α-l-arabinofuranosidase)** | | | | | | | | | |
|  | MYP_3646 | GH43 | ND | ND | ND | ND | OM | ND | NO |
|  | MYP_2165 | GH43, CBM6 | ND | ND | ND | ND | U | ND | YES |
|  | MYP_2166 | GH43, CBM6 | ND | ND | ND | ND | S | ND | YES |
|  | MYP_2103 | GH43_15, CBM6 | ND | ND | ND | ND | S | ND | YES |
|  | MYP_2107 | GH43, CBM6 | ND | ND | ND | ND | U | ND | YES |
| **GH53 (endo-β-1,4-galactanase)** | | | | | | | | | |
|  | MYP_3037 | GH53 | ND | ND | ND | ND | U | ND | YES |
| **GH57 (amylase)** | | | | | | | | | |
|  | MYP_329 | GH57 | 7.09 | ND | ND | ND | C | ND | NO |
| **GH65 (maltose phosphorylase)** | | | | | | | | | |
|  | MYP_2960 | GH65 | ND | ND | ND | ND | C | ND | NO |
| **GH74 (xyloglucanase)** | | | | | | | | | |
|  | MYP_4017 | GH74 | 6.54 | 6.93 | ND | ND | IM | ND | YES |
| **GH95 (α-1,2-l-fucosidase)** | | | | | | | | | |
|  | MYP_2162 | GH95, CBM6 | ND | ND | ND | ND | U | ND | YES |
| **GH97 (α-glucosidase)** | | | | | | | | | |
|  | MYP_3104 | GH97 | ND | ND | ND | ND | U | ND | NO |
| **GH108 (N-acetylmuramidase)** | | | | | | | | | |
|  | MYP_649 | GH108 | ND | ND | ND | ND | U | ND | NO |
| **GH133 (glycogen debranching enzyme)** | | | | | | | | | |
|  | MYP_4658 | GH133 | 7.00 | 7.73 | ND | ND | C | P | NO |
| **GH148 (β-1.3-glucanase)** | | | | | | | | | |
|  | MYP_3691 | GH148 | 7.3 | 7.7 | ND | 7.74 | P | P | NO |
| **CE1 (acetyl-xylan esterase, feruloyl esterase)** | | | | | | | | | |
|  | MYP_2108 | CE1, CBM6 | ND | ND | ND | ND | U | ND | YES |
|  | MYP_2163 | CE1, CBM6 | ND | ND | ND | ND | U | ND | YES |
| **CE2 (acetyl-xylan esterase)** | | | | | | | | | |
|  | MYP_3055 | CE2 | ND | ND | ND | ND | P | ND | NO |
| **CE4 (acetyl-xylan esterase, chitin deacetylase)** | | | | | | | | | |
|  | MYP_2179 | CE4 | ND | ND | ND | ND | C | ND | NO |
|  | MYP_3614 | GH8, CE4, CBM9 | ND | ND | ND | ND | OM | ND | YES |
|  | MYP_4267 | CE4 | ND | ND | ND | ND | C | ND | NO |
| **CE6 (acetyl-xylan esterase)** | | | | | | | | | |
|  | MYP_2109 | CE6, CBM6 | ND | ND | ND | ND | U | ND | YES |
|  | MYP_4086 | CE6, CBM9 | ND | ND | ND | ND | U | ND | YES |
| **CE8 (pectin methylesterase)** | | | | | | | | | |
|  | MYP_3868 | CE8, CBM35 | ND | ND | ND | ND | S | ND | YES |
| **CE11 (UDP-3-O-acyl N-acetylglucosamine deacetylase)** | | | | | | | | | |
|  | MYP_3791 | CE11 | 7.90 | 8.05 | ND | ND | C | OM | NO |
| **CE12 (pectin acetylesterase)** | | | | | | | | | |
|  | MYP_4087 | CE12, CBM35 | ND | ND | ND | ND | S | ND | YES |
|  | MYP_4088 | CE12, CBM35 | ND | ND | ND | ND | S | ND | YES |
| **CE15 (glucuronoyl esterase)** | | | | | | | | | |
|  | MYP_4345 | CE15, CBM9 | ND | ND | ND | ND | U | ND | YES |
|  |  |  |  |  |  |  |  |  |  |
| **CBM4 (xylan, β-1,3-glucan, β-1,3-1,4-glucan, β-1,6-glucan and amorphous cellulose)** | | | | | | | | | |
|  | MYP_2074 | GH10, CBM4 | ND | ND | ND | ND | S | ND | YES |
|  | MYP_4349 | GH16, CBM4 | ND | 6.91 | ND | ND | S | S | YES |
|  | MYP_4347 | GH8, CBM4 | ND | ND | ND | ND | S | ND | YES |
| **CBM6 (xylan)** | | | | | | | | | |
|  | MYP_458 | GH5, CBM6 | 6.88 | 6.63 | ND | ND | S | S | YES |
|  | MYP_1233 | CBM6 | ND | ND | ND | ND | OM | ND | YES |
|  | MYP_2104 | GH10, CBM6 | ND | ND | ND | ND | S | ND | YES |
|  | MYP_2109 | CE6, CBM6 | ND | ND | ND | ND | U | ND | YES |
|  | MYP_2103 | GH43_15, CBM6 | ND | ND | ND | ND | S | ND | YES |
|  | MYP_2108 | CE1, CBM6 | ND | ND | ND | ND | U | ND | YES |
|  | MYP_2165 | GH43, CBM6 | ND | ND | ND | ND | U | ND | YES |
|  | MYP_2163 | CE1, CBM6 | ND | ND | ND | ND | U | ND | YES |
|  | MYP_2082 | GH30_8, CBM6 | ND | ND | ND | ND | S | ND | YES |
|  | MYP_2107 | GH43, CBM6 | ND | ND | ND | ND | U | ND | YES |
|  | MYP_2162 | GH95, CBM6 | ND | ND | ND | ND | U | ND | YES |
|  | MYP_2166 | GH43, CBM6 | ND | ND | ND | ND | S | ND | YES |
|  | MYP_2462 | GH5_2, CBM6 | ND | ND | ND | ND | S | ND | YES |
|  | MYP_3455 | GH5_2, CBM6 | 7.46 | 5.86 | 6.62 | 6.62 | S | S | YES |
|  | MYP_3331 | GH10, CBM6 | ND | ND | ND | ND | S | ND | YES |
|  | MYP_2105 | CBM6, CBM51 | ND | ND | ND | ND | U | ND | YES |
| **CBM9 (xylan)** | | | | | | | | | |
|  | MYP_757 | CBM9 | 7.80 | 8.30 | ND | ND | IM | P | YES |
|  | MYP_1583 | GH8, CBM9 | ND | ND | ND | 7.93 | S | S | YES |
|  | MYP_2112 | GH8, CBM9 | 6.51 | 7.14 | ND | ND | OM | S | YES |
|  | MYP_2111 | GH11, CBM9 | ND | ND | ND | ND | S | ND | YES |
|  | MYP_3614 | GH8, CE4, CBM9 | ND | ND | ND | ND | OM | ND | YES |
|  | MYP_4086 | CE6, CBM9 | ND | ND | ND | ND | U | ND | YES |
|  | MYP_4345 | CE15, CBM9 | ND | ND | ND | ND | U | ND | YES |
|  | MYP_4127 | CBM9 | ND | ND | ND | ND | U | ND | YES |
|  | MYP_4198 | CBM9 | ND | ND | ND | ND | U | ND | NO |
| **CBM16 (cellulose/glucomannan)** | | | | | | | | | |
|  | MYP_3454 | GH16, CBM16 | ND | ND | ND | ND | S | ND | YES |
| **CBM35 (xylan)** | | | | | | | | | |
|  | MYP_4095 | PL11_1, CBM35 | ND | ND | ND | ND | S | ND | YES |
|  | MYP_4094 | PL11, CBM35 | ND | ND | ND | ND | S | ND | YES |
|  | MYP_4610 | GH26, CBM35 | ND | 7.17 | ND | ND | U | ND | YES |
|  | MYP_3868 | CE8, CBM35 | ND | ND | ND | ND | S | ND | YES |
|  | MYP_4087 | CE12, CBM35 | ND | ND | ND | ND | S | ND | YES |
|  | MYP_4088 | CE12, CBM35 | ND | ND | ND | ND | S | ND | YES |
| **CBM50 (peptidoglycan)** | | | | | | | | | |
|  | MYP_105 | GH23, CBM50 | ND | ND | ND | ND | C | ND | NO |
|  | MYP_14 | CBM50 | 7.94 | 7.56 | ND | ND | U | OM | NO |
|  | MYP_971 | GH23, CBM50 | ND | ND | ND | ND | C | ND | NO |
|  | MYP_2642 | CBM50 | ND | 6.69 | ND | ND | IM | ND | NO |
|  | MYP_4281 | CBM50 | 8.06 | 7.98 | ND | ND | U | S | NO |
|  | MYP_4307 | GH23, CBM50 | ND | ND | ND | ND | S | ND | NO |
| **CBM51 (galactose, blood group antigens)** | | | | | | | | | |
|  | MYP_2105 | CBM6, CBM51 | ND | ND | ND | ND | U | ND | YES |
| **CBM57 (unknown)** | | | | | | | | | |
|  | MYP_550 | CBM57 | ND | 7.50 | ND | ND | U | ND | YES |
|  | MYP_563 | CBM57 | 7.42 | 7.47 | ND | 7.57 | IM | P | YES |
|  | MYP_1768 | CBM57 | ND | ND | ND | ND | U | ND | YES |
|  | MYP_2011 | CBM57 | ND | ND | ND | ND | U | ND | YES |
| **CBM62 (galactose)** | | | | | | | | | |
|  | MYP_1693 | CBM62 | ND | ND | ND | ND | S | ND | YES |
|  | MYP_4107 | CBM62 | ND | ND | ND | ND | U | ND | YES |
| **PL1 (pectin lyase)** | | | | | | | | | |
|  | MYP_4611 | PL1 | ND | ND | ND | ND | S | ND | YES |
|  | MYP_4344 | PL1 | ND | ND | ND | ND | S | ND | YES |
| **PL9 (pectate lyase)** | | | | | | | | | |
|  | MYP_3575 | PL9_1 | ND | ND | ND | ND | S | ND | YES |
| **PL11 (rhamnogalacturonan lyase)** | | | | | | | | | |
|  | MYP_4095 | PL11_1, CBM35 | ND | ND | ND | ND | S | ND | YES |
|  | MYP_4094 | PL11, CBM35 | ND | ND | ND | ND | S | ND | YES |
| **PL14 (alginate lyase)** | | | | | | | | | |
|  | MYP_4081 | PL14_3 | ND | ND | ND | ND | U | ND | YES |
| **GT2 (β-glycans)** | | | | | | | | | |
|  | MYP_44 | GT2 | ND | ND | ND | ND | C | ND | NO |
|  | MYP_228 | GT2 | ND | ND | ND | ND | C | ND | NO |
|  | MYP_556 | GT2 | ND | ND | ND | ND | IM | ND | NO |
|  | MYP_557 | GT2 | ND | 7.31 | ND | ND | IM | OM | NO |
|  | MYP_564 | GT2 | ND | ND | ND | ND | C | ND | NO |
|  | MYP_573 | GT2 | ND | ND | ND | ND | C | ND | NO |
|  | MYP_1011 | GT2 | ND | ND | ND | ND | C | ND | NO |
|  | MYP_1012 | GT2 | ND | ND | ND | ND | C | ND | NO |
|  | MYP_1013 | GT2 | ND | ND | ND | ND | OM | ND | NO |
|  | MYP_1014 | GT2 | ND | ND | ND | ND | C | ND | NO |
|  | MYP_1046 | GT4, GT2 | ND | ND | ND | ND | OM | ND | NO |
|  | MYP_1047 | GT2 | ND | ND | ND | ND | C | ND | NO |
|  | MYP_1249 | GT2 | ND | ND | ND | ND | IM | ND | NO |
|  | MYP_1276 | GT2 | ND | ND | ND | ND | C | ND | NO |
|  | MYP_1281 | GT2 | ND | ND | ND | ND | C | ND | NO |
|  | MYP_1291 | GT2 | ND | ND | ND | ND | C | ND | NO |
|  | MYP_1316 | GT2 | ND | ND | ND | ND | IM | ND | NO |
|  | MYP_1673 | GT2 | ND | ND | ND | ND | C | ND | NO |
|  | MYP_1684 | GT2 | ND | ND | ND | ND | IM | ND | NO |
|  | MYP_2009 | GT2 | ND | ND | ND | ND | IM | ND | NO |
|  | MYP_2040 | GT2 | 7.40 | 7.62 | ND | ND | C | OM | NO |
|  | MYP_2178 | GT2 | ND | ND | ND | ND | IM | ND | NO |
|  | MYP_2239 | GT2 | ND | ND | ND | ND | C | ND | NO |
|  | MYP_2477 | GT2 | ND | ND | ND | ND | IM | ND | NO |
|  | MYP_2483 | GT2 | ND | ND | ND | ND | C | ND | NO |
|  | MYP_2489 | GT2 | ND | ND | ND | ND | IM | ND | NO |
|  | MYP_2490 | GT2 | ND | ND | ND | ND | C | ND | NO |
|  | MYP_3094 | GT2 | ND | ND | ND | ND | C | ND | NO |
|  | MYP_3177 | GT2 | ND | ND | ND | ND | C | ND | NO |
|  | MYP_3228 | GT2 | ND | ND | ND | ND | IM | ND | NO |
|  | MYP_3390 | GT2 | ND | ND | ND | ND | OM | ND | NO |
|  | MYP_3391 | GT2 | ND | ND | ND | ND | IM | ND | NO |
|  | MYP_3497 | GT2 | ND | 6.82 | ND | ND | C | ND | NO |
|  | MYP_3817 | GT2 | ND | ND | ND | ND | C | ND | NO |
|  | MYP_3938 | GT2 | ND | ND | ND | ND | C | ND | NO |
|  | MYP_3941 | GT2 | ND | ND | ND | ND | C | ND | NO |
|  | MYP_4271 | GT2 | ND | ND | ND | ND | IM | ND | NO |
|  | MYP_4449 | GT2 | ND | ND | ND | ND | IM | ND | NO |
|  | MYP_4494 | GT2 | ND | ND | ND | ND | C | ND | NO |
| **GT3 (glycogen synthase)** | | | | | | | | | |
|  | MYP_760 | GT3 | 7.68 | 8.24 | 7.34 | 7.45 | C | C | NO |
| **GT4 (α-glycans)** | | | | | | | | | |
|  | MYP_330 | GT4, GT5 | 7.78 | 6.70 | ND | ND | C | OM | NO |
|  | MYP_486 | GT4 | ND | ND | ND | ND | C | ND | NO |
|  | MYP_490 | GT4 | ND | ND | ND | ND | C | ND | NO |
|  | MYP_560 | GT4 | 6.76 | 6.59 | ND | ND | OM | OM | NO |
|  | MYP_985 | GT4 | 7.25 | 7.15 | ND | ND | C | C | NO |
|  | MYP_1016 | GT4 | ND | ND | ND | ND | C | ND | NO |
|  | MYP_1017 | GT4 | ND | ND | ND | ND | C | ND | NO |
|  | MYP_1046 | GT4, GT2 | ND | ND | ND | ND | OM | ND | NO |
|  | MYP_1282 | GT4 | ND | ND | ND | ND | C | ND | NO |
|  | MYP_1284 | GT4 | ND | ND | ND | ND | C | ND | NO |
|  | MYP_1285 | GT4 | ND | ND | ND | ND | C | ND | NO |
|  | MYP_1289 | GT4 | ND | ND | ND | ND | C | ND | NO |
|  | MYP_1290 | GT4 | ND | ND | ND | ND | C | ND | NO |
|  | MYP_1293 | GT4 | ND | ND | ND | ND | C | ND | NO |
|  | MYP_1644 | GT4 | ND | ND | ND | ND | C | ND | NO |
|  | MYP_2180 | GT4 | ND | 6.87 | ND | ND | C | OM | NO |
|  | MYP_2360 | GT4 | 6.72 | 7.16 | ND | ND | C | ND | NO |
|  | MYP_2361 | GT4 | ND | ND | ND | ND | C | ND | NO |
|  | MYP_2422 | GT4 | ND | ND | ND | ND | C | ND | NO |
|  | MYP_2493 | GT4 | ND | ND | ND | ND |  | ND | NO |
|  | MYP_3185 | GT4 | ND | ND | ND | ND | C | ND | NO |
|  | MYP_3186 | GT4 | ND | ND | ND | ND | C | ND | NO |
|  | MYP_3279 | GT4 | ND | ND | ND | ND | C | ND | NO |
|  | MYP_3393 | GT4 | 7.22 | 6.14 | ND | ND | C | OM | NO |
|  | MYP_3652 | GT4 | ND | ND | ND | ND | C | ND | NO |
|  | MYP_3742 | GT4 | ND | ND | ND | ND | C | ND | NO |
|  | MYP_4038 | GT4 | ND | ND | ND | ND | C | ND | NO |
|  | MYP_4234 | GT4 | ND | ND | ND | ND | C | ND | NO |
|  | MYP_4319 | GT4 | ND | ND | ND | ND | C | ND | NO |
| **GT9 (lipopolysaccharide N-acetylglucosaminyltransferase)** | | | | | | | | | |
|  | MYP_1186 | GT9 | ND | ND | ND | ND | C | ND | NO |
|  | MYP_1410 | GT9 | ND | ND | ND | ND | P | ND | NO |
|  | MYP_2362 | GT9 | ND | ND | ND | ND | C | ND | NO |
|  | MYP_3183 | GT9 | ND | ND | ND | ND | C | ND | NO |
|  | MYP_3184 | GT9 | ND | ND | ND | ND | C | ND | NO |
|  | MYP_3190 | GT9 | ND | ND | ND | ND | C | ND | NO |
| **GT11 (GDP-l-Fuc: galactoside α-1,2-l-fucosyltransferase)** | | | | | | | | | |
|  | MYP_1010 | GT11 | ND | ND | ND | ND | C | ND | NO |
| **GT19 (lipid-A-disaccharide synthase)** | | | | | | | | | |
|  | MYP_4644 | GT19 | ND | ND | ND | ND | C | ND | NO |
| **GT20 (trehalose-6-P phosphatase)** | | | | | | | | | |
|  | MYP_2116 | GT20 | ND | ND | ND | ND | C | ND | NO |
|  | MYP_3131 | GT20 | 6.69 | 7.32 | ND | ND | C | OM | NO |
|  | MYP_3690 | GT20 | 7.32 | 7.00 | ND | ND | C | OM | NO |
| **GT25 (β-1,2-glucosyltransferase)** | | | | | | | | | |
|  | MYP_631 | GT25 | ND | ND | ND | ND | C | ND | NO |
| **GT27 (polypeptide α-N-acetylgalactosaminyltransferase)** | | | | | | | | | |
|  | MYP_507 | GT27 | ND | ND | ND | ND | C | ND | NO |
| **GT28 (1,2-diacylglycerol 3-β-galactosyltransferase)** | | | | | | | | | |
|  | MYP_2014 | GT28 | ND | ND | ND | ND | C | ND | NO |
|  | MYP_4221 | GT28 | 6.95 | 7.37 | ND | ND | IM | OM | NO |
| **GT30 (CMP-β-KDO: α-3-deoxy-d-manno-octulosonic-acid (KDO) transferase)** | | | | | | | | | |
|  | MYP_230 | GT30 | ND | ND | ND | ND | C | ND | NO |
| **GT35 (glycogen or starch phosphorylase)** | | | | | | | | | |
|  | MYP_470 | GT35 | 7.93 | 8.03 | ND | 7.42 | C | OM | NO |
| **GT51 (murein polymerase)** | | | | | | | | | |
|  | MYP_467 | GT51 | ND | ND | ND | ND | OM | ND | NO |
|  | MYP_923 | GT51 | ND | ND | ND | ND | OM | ND | NO |
|  | MYP_940 | GT51 | 6.90 | 7.68 | ND | ND | OM | OM | NO |
|  | MYP_1359 | GT51 | ND | 7.02 | ND | ND | OM | OM | NO |
|  | MYP_3589 | GT51 | 6.87 | 7.00 | ND | ND | OM | OM | NO |
|  | MYP_4680 | GT51 | 6.90 | 7.81 | ND | ND | P | P | NO |
|  | MYP_4686 | GT51 | ND | ND | ND | ND | C | ND | NO |
| **GT83 (undecaprenyl phosphate-α-l-Ara4N: 4-amino-4-deoxy-β-l-arabinosyltransferase)** | | | | | | | | | |
|  | MYP_1682 | GT83 | ND | ND | ND | ND | IM | ND | NO |
|  | MYP_2039 | GT83 | ND | ND | ND | ND | IM | ND | NO |
|  | MYP_4447 | GT83 | ND | ND | ND | ND | IM | ND | NO |
| **GT105 (Dol-P-Man : protein α-mannosyltransferase)** | | | | | | | | | |
|  | MYP_2527 | GT105 | ND | ND | ND | ND | IM | ND | NO |
