# Supplementary material for: Proteomic Dissection of the Cellulolytic Machineries Used by Soil-Dwelling Bacteroidetes
Source: mSystems. 2018 Nov 20;3(6):e00240-18. doi: 10.1128/mSystems.00240-18 (PMC6247017; doi:10.1128/mSystems.00240-18)
Supplement: TABLE S3 [file sys006182297st3.docx]

**Table S3**: Percent total log_10_ LFQ intensities of the cell localization markers in both *C. hutchinsonii* and *S. myxococcoides*. ompA (outer membrane protein A) - outer membrane, ptsS (phosphate ABC transporter phosphate-binding protein) – periplasm, nuoL (NADH-quinone oxidoreductase subunit L) – inner membrane.

|  |  | **Average % total LFQ intensity** | | | | |
| --- | --- | --- | --- | --- | --- | --- |
|  | **Locus Tag** | **Cyt** | **IM** | **Per** | **OM** | **Sec** |
| **ompA** | CHU_1710 | n.d. | n.d. | n.d. | 0.0320 | n.d. |
| **pstS** | CHU_3818 | 0.2237 | 0.7673 | 1.1052 | 0.2088 | 0.1762 |
| **nuoL** | CHU_1371 | 0.0086 | 0.0265 | n.d. | 0.0040 | 0.0030 |
|  |  |  |  |  |  |  |
| **ompA** | MYP_505 | 0.5423 | 2.2857 | 2.3683 | 3.3486 | 2.7250 |
| **pstS** | MYP_603 | 0.0037 | 0.0138 | 0.0390 | 0.0057 | 0.0174 |
| **nuoL** | MYP_729 | n.d. | 0.0068 | n.d. | n.d. | n.d. |

n.d. – Not detected
