# Supplementary material for: Proteomic Dissection of the Cellulolytic Machineries Used by Soil-Dwelling Bacteroidetes
Source: mSystems. 2018 Nov 20;3(6):e00240-18. doi: 10.1128/mSystems.00240-18 (PMC6247017; doi:10.1128/mSystems.00240-18)
Supplement: TABLE S4 [file sys006182297st4.docx]

**Table S4**

|  | **Locus Tag** | **Early Filter Paper** | **Late Filter Paper** | **Early Pectin** | **Late Pectin** | **Pred. Local** | **Local** |
| --- | --- | --- | --- | --- | --- | --- | --- |
| **gldA** | CHU_1545 | ND | ND | ND | ND | C | ND |
| **gldB** | CHU_3691 | 6.14 | 6.14 | ND | 6.09 | IM | IM |
| **gldD** | CHU_3683 | ND | ND | ND | ND | P | ND |
| **gldF** | CHU_1546 | ND | ND | ND | ND | IM | ND |
| **gldG** | CHU_1547 | 5.88 | 5.37 | ND | ND | C | ND |
| **gldH** | CHU_0291 | 5.63 | ND | ND | ND | OM | ND |
| **gldJ** | CHU_3494 | 6.84 | 7.08 | 5.77 | 5.16 | S | OM |
| **gldK** | CHU_0171 | 7.03 | 7.44 | 6.62 | 6.4 | S | OM |
| **gldL** | CHU_0172 | 7.09 | 6.76 | 6.01 | 5.67 | S | IM |
| **gldM** | CHU_0173 | 7.67 | 7.56 | 6.86 | 6.07 | P | IM |
| **gldM** | CHU_2853 | ND | ND | ND | ND | OM | ND |
| **gldM** | CHU_3492 | ND | ND | ND | ND | P | ND |
| **gldN** | CHU_0174 | 7.38 | 7.16 | 5.91 | 5.88 | C | IM |
| **gldN** | CHU_2610 | ND | ND | ND | ND | IM | ND |
| **sprA** | CHU_0291 | ND | ND | ND | ND | OM | ND |
| **sprE** | CHU_0177 | ND | ND | ND | ND | OM | ND |
| **sprT** | CHU_3070 | ND | ND | ND | ND | C | ND |
| **sprB** | CHU_2225 | 4.6 | 4.56 | ND | ND | S | ND |
| **sprP** | CHU_0170 | 6.41 | 6.37 | 5.56 | ND | IM | IM |
| **sprP** | CHU_3434 | 5.37 | 5.89 | ND | ND | OM | OM |
