# Supplementary material for: Proteomic Dissection of the Cellulolytic Machineries Used by Soil-Dwelling Bacteroidetes
Source: mSystems. 2018 Nov 20;3(6):e00240-18. doi: 10.1128/mSystems.00240-18 (PMC6247017; doi:10.1128/mSystems.00240-18)
Supplement: TABLE S5 [file sys006182297st5.docx]

**Table S5**

|  | **Locus Tag** | **Early Filter Paper** | **Late Filter Paper** | **Early Pectin** | **Late Pectin** | **Pred. Local** | **Local** |
| --- | --- | --- | --- | --- | --- | --- | --- |
| **gldB** | MYP_1643 | 7.05 | 7.19 | 6.43 | 6.21 | IM | IM, OM |
| **gldD** | MYP_1634 | 7.17 | 6.77 | 6.22 | 6.24 | P | OM |
| **gldF** | MYP_2215 | ND | ND | ND | ND | IM | ND |
| **gldG** | MYP_2214 | 7.46 | 7.62 | 6.65 | 6.3 | OM | IM |
| **gldH** | MYP_4535 | 6.3 | 6.5 | 6.2 | 6.3 | P | ND |
| **gldJ** | MYP_1657 | 8.9 | 9.24 | 7.19 | 7.9 | OM | OM |
| **gldK** | MYP_935 | 8.85 | 9.25 | 7.9 | 8.15 | S | OM |
| **gldL** | MYP_936 | 8.46 | 8.11 | 8.03 | 7.36 | P | P |
| **gldM** | MYP_937 | 9.25 | 8.76 | 8.61 | 7.75 | P | P |
| **gldM** | MYP_1963 | ND | ND | ND | ND | P | ND |
| **gldN** | MYP_938 | 9.21 | 9.56 | 8.38 | 8.35 | IM | OM |
| **sprE** | MYP_941 | 7.24 | 7.5 | 7.42 | 6.27 | OM | OM |
| **sprT** | MYP_4441 | ND | ND | ND | ND | C | ND |
| **sprB** | MYP_3087 | 6.32 | 6.32 | 6.37 | 6.33 | S | ND |
