# Supplementary material for: Proteomic Dissection of the Cellulolytic Machineries Used by Soil-Dwelling Bacteroidetes
Source: mSystems. 2018 Nov 20;3(6):e00240-18. doi: 10.1128/mSystems.00240-18 (PMC6247017; doi:10.1128/mSystems.00240-18)
Supplement: TABLE S6 [file sys006182297st6.docx]

**Table S6**

| **Locus tag** | **Log_10_ LFQ intensity** | **Pfam** | **Mol. weight [kDa]** | **Local** | **T9SS** |
| --- | --- | --- | --- | --- | --- |
| ***C. hutchinsonii*** | | | | | |
| CHU_2679 | **8.46** |  | 65.5 | OM | No |
| CHU_0007 | **7.75** |  | 27.442 | S | No |
| CHU_3732 | **7.61** | DUF1597 | 44.08 | S | No |
| CHU_1277 | **7.59** |  | 65.456 | S | No |
| CHU_3459 | **7.59** |  | 33.71 | OM | No |
| CHU_3384 | **7.33** |  | 33.789 | S | No |
| CHU_1276 | **7.22** |  | 85.25 | OM | No |
| CHU_0986 | **7.19** |  | 26.887 | S | No |
| CHU_2590 | **7.11** |  | 34.1 | OM | No |
| CHU_3300 | **7.10** |  | 30.096 | S | No |
| CHU_0090 | **7.09** |  | 49.016 | S | No |
| CHU_1935 | **7.04** |  | 26.24 | OM | No |
| CHU_3220 | **7.01** |  | 198.92 | S | No |
| CHU_0344 | **6.93** |  | 95.21 | OM | Yes |
| CHU_1253 | **6.91** |  | 49.95 | S | No |
| CHU_2632 | **6.91** |  | 52.927 | S | No |
| CHU_3816 | **6.90** | DUF2490 | 39.309 | S | No |
| CHU_1366 | **6.89** | DUF4480;Plug;TonB_dep_Rec | 91.167 | S | No |
| CHU_1818 | **6.88** |  | 24.708 | S | No |
| CHU_0247 | **6.87** |  | 25.745 | S | No |
| CHU_3709 | **6.84** | DUF4480;Plug;TonB_dep_Rec | 87.635 | S | No |
| CHU_3346 | **6.84** |  | 32.886 | S | No |
| CHU_0788 | **6.76** |  | 35.816 | S | No |
| CHU_3437 | **6.76** |  | 248.1 | S | Yes |
| CHU_1278 | **6.73** |  | 41.48 | OM | No |
| CHU_3238 | **6.66** |  | 42.775 | S | No |
| CHU_2868 | **6.63** | DUF2911 | 32.552 | S | No |
| CHU_0198 | **6.61** | DUF4480;Plug;TonB_dep_Rec | 95.67 | S | No |
| CHU_1960 | **6.61** | DUF3078 | 35.63 | S | No |
| CHU_3711 | **6.58** |  | 26.745 | S | No |
| CHU_0878 | **6.57** |  | 108.86 | S | Yes |
| CHU_0910 | **6.57** |  | 57.83 | OM | No |
| CHU_2333 | **6.56** | DUF3897 | 22.22 | OM | No |
| CHU_1936 | **6.54** |  | 24.279 | S | No |
| CHU_0922 | **6.54** |  | 82.241 | S | Yes |
| CHU_0361 | **6.53** |  | 94.196 | S | Yes |
| CHU_1860 | **6.49** |  | 35.603 | S | No |
| CHU_1255 | **6.46** |  | 49.163 | S | No |
| CHU_3021 | **6.44** |  | 26.108 | S | Yes |
| CHU_3779 | **6.44** |  | 21.705 | S | No |
| CHU_1279 | **6.43** |  | 42.421 | S | No |
| CHU_0268 | **6.43** |  | 66.411 | S | Yes |
| CHU_0047 | **6.38** |  | 18.4 | OM | No |
| CHU_2472 | **6.37** |  | 26.32 | OM | No |
| CHU_2208 | **6.32** |  | 53.68 | OM | No |
| CHU_2210 | **6.28** | DUF3341 | 19.163 | S | No |
| CHU_0335 | **6.25** |  | 62.38 | OM | No |
| CHU_0170 | **6.25** | DUF3308 | 38.88 | OM | No |
| CHU_1521 | **6.24** |  | 198.74 | S | No |
| CHU_3618 | **6.24** |  | 40.675 | S | Yes |
| ***S. myxococcoides*** | | | | | |
| MYP_3502 | **9.50** |  | 33.966 | OM | No |
| MYP_4704 | **9.19** | OMP_b-brl_2 | 24.619 | OM | No |
| MYP_925 | **9.11** |  | 24.854 | S | No |
| MYP_74 | **9.03** |  | 33.798 | S | No |
| MYP_1253 | **8.98** |  | 28.067 | OM | No |
| MYP_4241 | **8.95** |  | 78.991 | OM | No |
| MYP_4242 | **8.90** |  | 66.825 | S | No |
| MYP_2344 | **8.89** |  | 34.482 | OM | No |
| MYP_4697 | **8.79** |  | 44.486 | S | No |
| MYP_3562 | **8.73** |  | 110.96 | OM | No |
| MYP_3603 | **8.73** | OMP_b-brl | 23.836 | OM | No |
| MYP_4244 | **8.68** |  | 68.375 | OM | No |
| MYP_4440 | **8.65** |  | 110.38 | OM | No |
| MYP_3127 | **8.62** |  | 34.286 | OM | No |
| MYP_4853 | **8.61** |  | 38.132 | S | No |
| MYP_782 | **8.47** | DUF3897 | 22.998 | S | No |
| MYP_4833 | **8.46** | OMP_b-brl | 35.394 | OM | No |
| MYP_538 | **8.39** |  | 29.705 | S | Yes |
| MYP_147 | **8.33** |  | 45.425 | OM | No |
| MYP_4452 | **8.32** |  | 183.98 | OM | No |
| MYP_1051 | **8.31** |  | 59.999 | OM | No |
| MYP_3406 | **8.31** | DUF4468 | 20.518 | S | No |
| MYP_934 | **8.23** | DUF3308 | 39.459 | S | No |
| MYP_413 | **8.21** | DUF4480;Plug | 90.216 | S | No |
| MYP_3893 | **8.20** |  | 40.435 | S | No |
| MYP_1564 | **8.18** |  | 46.095 | S | No |
| MYP_4674 | **8.17** |  | 21.109 | OM | No |
| MYP_3555 | **8.15** |  | 25.46 | OM | No |
| MYP_921 | **8.14** |  | 40.877 | S | No |
| MYP_1962 | **8.12** |  | 45.954 | OM | No |
| MYP_2911 | **8.10** |  | 392.94 | S | No |
| MYP_1744 | **8.10** | DUF4369;Thioredoxin_8 | 53.796 | S | No |
| MYP_4176 | **8.06** |  | 34.238 | OM | No |
| MYP_2456 | **8.06** | DUF4480;Plug;TonB_dep_Rec | 109.94 | S | No |
| MYP_579 | **8.05** | DUF853 | 57.14 | OM | No |
| MYP_4542 | **8.02** | DUF2723 | 111.89 | OM | No |
| MYP_1521 | **8.01** | DUF4480;Plug;TonB_dep_Rec | 92.48 | OM | No |
| MYP_2646 | **7.99** | DUF349 | 72.374 | OM | No |
| MYP_1375 | **7.98** |  | 56.566 | OM | No |
| MYP_3107 | **7.98** |  | 69.367 | S | Yes |
| MYP_3867 | **7.95** | OMP_b-brl | 22.484 | S | No |
| MYP_2894 | **7.93** |  | 35.835 | S | No |
| MYP_31 | **7.92** |  | 19.974 | OM | No |
| MYP_1642 | **7.92** | OMP_b-brl_2 | 21.917 | OM | No |
| MYP_1364 | **7.91** | DUF4468 | 19.951 | OM | No |
| MYP_994 | **7.90** |  | 57.111 | S | No |
| MYP_897 | **7.88** |  | 31.848 | S | No |
| MYP_3075 | **7.87** | DUF2911 | 30.809 | S | No |
| MYP_3549 | **7.85** | DUF4255 | 20.579 | OM | No |
| MYP_2725 | **7.82** |  | 24.288 | S | No |
